# Alzheimer’s Disease as a Multi-Layer Network Disorder: A Systems Biology Framework Integrating Multi-Omics Mechanisms

**DOI:** 10.3390/biomedicines14081823

**Published:** 2026-08-13

**Authors:** Muhammed Alzweiri, Ahmed S. A. Ali Agha, Nidal A. Qinna, Ghayda’ AlDabet, Thaqif El Khassawna, Talal Aburjai

**Affiliations:** 1 Department of Pharmaceutical Sciences, School of Pharmacy, The University of Jordan, Amman 11942, Jordan; thaqif.elkhassawna@chiru.med.uni-giessen.de (T.E.K.); aburjai@ju.edu.jo (T.A.); 2 Department of Pharmacy, Pharmacological and Diagnostic Research Centre, Al-Ahliyya Amman University, Amman 19111, Jordan; asat3u@gmail.com; 3 Faculty of Pharmacy and Medical Sciences, University of Petra, Amman 11196, Jordan; nqinna@uop.edu.jo; 4 University of Petra Pharmaceutical Center (UPPC), Faculty of Pharmacy and Medical Sciences, University of Petra, Amman 11196, Jordan; ghayda.aldabet@uop.edu.jo; 5 Experimental Trauma Surgery, Faculty of Medicine, Justus-Liebig-University of Giessen, 35392 Giessen, Germany

**Keywords:** Alzheimer’s disease, network disorder, multi-omics, disease progression, biomarker heterogeneity, synaptic dysfunction, neuroinflammation, mitochondrial dysfunction

## Abstract

Despite substantial progress in biomarker discovery and multi-omics profiling, several features of Alzheimer’s disease (AD), including prolonged compensated states, heterogeneous clinical trajectories, and marked stage-dependent therapeutic responses, remain difficult to integrate into a single mechanistic framework. In this review, we propose an integrative and testable conceptual framework that reframes AD as a single, progressive multi-layer network disorder whose dynamics arise from hierarchical constraint propagation and progressive loss of cross-scale coordination. Integrating evidence from human genetics, epigenomics, transcriptomics, proteomics, metabolomics, spatial biology, connectomics, and longitudinal biomarker studies, we examine how molecular, cellular, and circuit-level processes interact over time to shape disease progression. Within this framework, different omics measurements are interpreted as complementary representations of disease-related changes, rather than as independent molecular signatures. Disease progression reflects the gradual convergence of immune, metabolic, proteostatic, cytoskeletal, and synaptic stress, with overt cognitive impairment emerging when compensatory capacity is exceeded, producing threshold-like network destabilization. By explicitly linking biological scale, temporal hierarchy, and network structure, this synthesis extends prior network-medicine, connectomic, and multi-omics approaches into a testable framework for state-aware stratification, integrative analysis, and stage-appropriate therapeutic investigation in AD.

## 1. Introduction

Alzheimer’s disease (AD) is now defined biologically by amyloid-β and tau biomarkers rather than solely by clinical syndromes [1], a shift formalized in the 2018 National Institute on Aging–Alzheimer’s Association framework [2] and expanded in the 2024 diagnostic criteria [3]. This shift has enabled earlier identification along the disease continuum and has supported biomarker-guided stratification in clinical trials. However, while these frameworks operationalize pathology, they do not mechanistically explain how cognitive decline emerges from interacting molecular, cellular, and systems-level processes, nor why comparable amyloid and tau burdens yield divergent clinical trajectories.

Historically, AD pathogenesis has been organized most prominently around the amyloid cascade hypothesis [4,5,6]. However, several non-mutually exclusive hypotheses emphasize tau propagation and phase transition, neuroimmune activation, mitochondrial and redox failure, neurovascular dysfunction, and endolysosomal/proteostatic collapse [7,8,9,10,11,12]. These mechanisms are better viewed as interacting constraints than as mutually exclusive alternatives, because each can alter the threshold at which amyloid and tau pathology are translated into synaptic and clinical dysfunction. Earlier single-target interventions often produced biological effects without durable clinical benefit, particularly after substantial symptomatic progression [5,13].

Lecanemab and donanemab produce modest slowing of decline in selected patients with early symptomatic, biomarker-confirmed AD [14,15], but they also introduce clinically important treatment toxicity. In their pivotal trials, amyloid-related imaging abnormalities with edema/effusion (ARIA-E) occurred in 12.6% of lecanemab-treated and 24.0% of donanemab-treated participants; symptomatic ARIA-E occurred in 2.8% and 6.1%, respectively, and infusion-related reactions were also common [14,15]. Amyloid-related imaging abnormalities with hemosiderin deposition (ARIA-H) and intracerebral hemorrhage can be serious and, rarely, fatal. Risk is higher in apolipoprotein E ε4 carriers, particularly homozygotes; appropriate use therefore requires individualized benefit–risk assessment, baseline and surveillance magnetic-resonance imaging, and caution in patients with substantial bleeding risk or probable cerebral amyloid angiopathy [16,17].

The limited magnitude and stage dependence of single-target benefit, together with biological heterogeneity and treatment risk, have motivated multi-target-directed ligands and systems-oriented strategies [18,19,20,21,22]. A systems framework does not presume that simultaneous inhibition of multiple pathways is inherently superior. Rather, it seeks to identify which module or cross-module interface is dominant in a given disease state and when a mechanism-specific, sequential, or combination strategy is biologically justified.

Converging evidence from connectomics, single-cell profiling, and large-scale multi-omic studies further extends these earlier multi-target and systems-oriented concepts, indicating that AD reflects coordinated perturbations across molecular interaction networks, cell-type ecosystems, and large-scale brain circuits [23,24,25]. Amyloid and tau accumulation can coexist with prolonged compensation, suggesting that pathology is buffered until system-level resilience erodes [26,27]. Age-related decline in proteostasis, autophagic flux, mitochondrial bioenergetics, and immune regulation further supports a model in which chronic stress progressively narrows buffering capacity, eventually precipitating a critical transition toward widespread dysfunction [28]. Importantly, no single omics modality captures this destabilization in isolation; rather, genomic, epigenomic, transcriptomic, proteomic, metabolomic, and spatial layers represent complementary projections of an underlying systems-level process that unfolds across scales.

These insights align with principles from network medicine and complex systems theory, which conceptualize disease as perturbation of interconnected modules rather than failure of isolated pathways. In such architectures, robustness coexists with fragility: distributed stress may be tolerated, but deterioration of modular independence and hub integrity can trigger disproportionate system-level effects [29,30]. This perspective provides a conceptual bridge between molecular pathology, cellular reprogramming, and circuit-level disintegration.

Existing approaches address complementary but distinct parts of AD organization. Network medicine maps disease-associated genes and proteins onto interactomes to identify modules, hubs, and candidate regulators [23,24,31,32]; connectome-based models describe the propagation and failure of large-scale brain networks [25,29,30]; and multi-omics integration pipelines recover shared latent factors or molecular subtypes without necessarily assigning cross-scale directionality [33,34]. The present framework does not replace these approaches. It connects them through three operational commitments. First, hierarchical constraint propagation means that the state of one biological layer changes the range or probability of states accessible to the next layer, rather than merely correlating with it. Second, cross-scale buffering denotes redundant or adaptive regulatory, metabolic, synaptic, and network routes that preserve function despite pathology and whose loss can be estimated longitudinally. Third, state-aware stratification classifies individuals by a joint configuration of modules, layers, and their coupling rather than by a single biomarker or clinical label. The specific advance is therefore an explicitly longitudinal, cross-scale hypothesis: clinical deterioration becomes more likely as accessible adaptive states contract, module redundancy falls, and coupling among formerly semi-autonomous modules increases.

**Figure 1:**
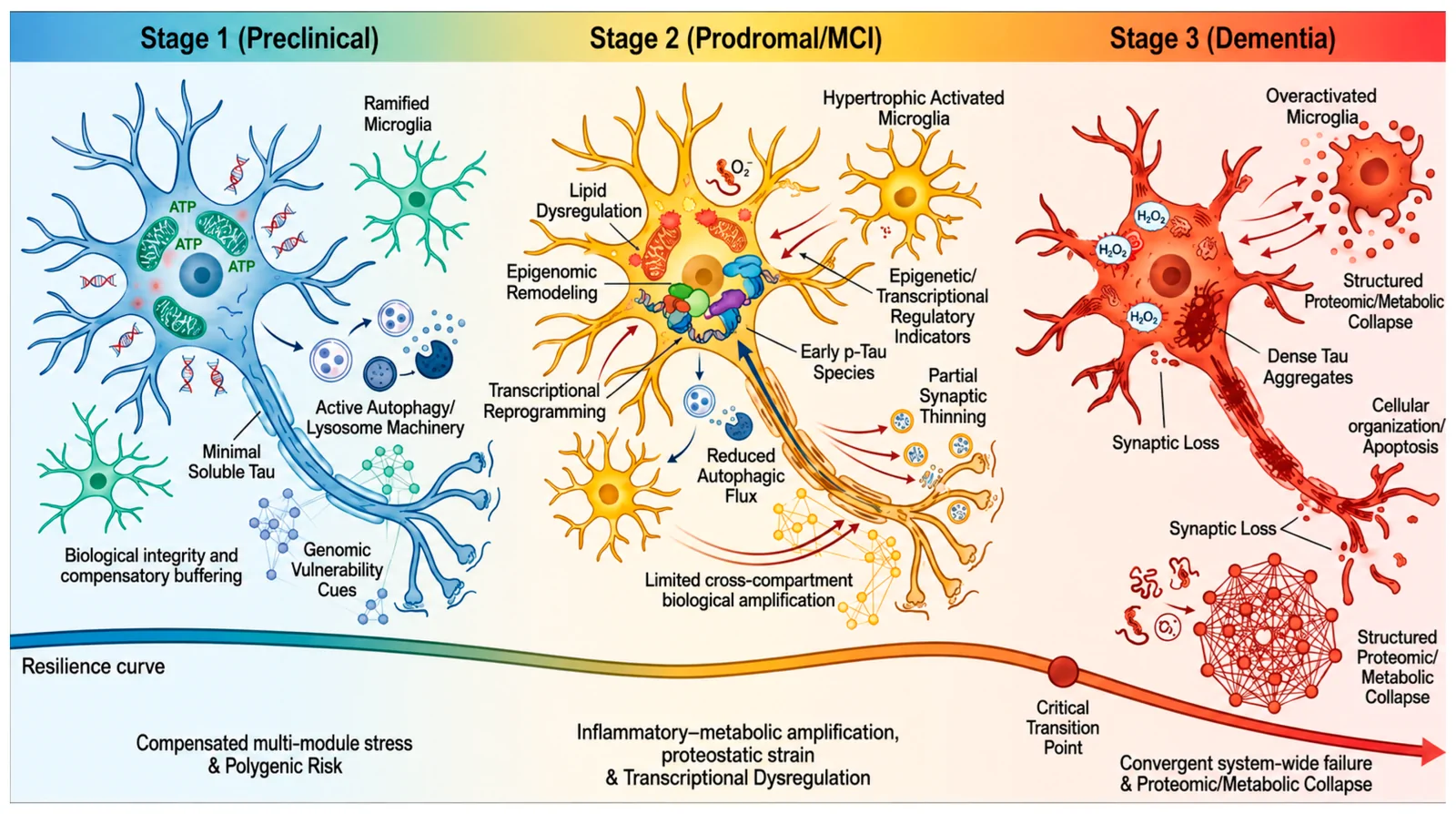
Clinically aligned conceptual states of progressive multi-layer network destabilization in AD. Preclinical AD (2024 Alzheimer’s Association clinical stages 1–2) is modeled as compensated multi-module stress; prodromal/MCI due to AD (stage 3) as inflammatory–metabolic amplification with rising cross-module coupling; and AD dementia (stages 4–6) as decompensated synaptic and connectomic failure. These composite states are hypotheses for longitudinal validation and are not presented as established treatment algorithms.

In this review, we synthesize convergent evidence across genomic, epigenomic, transcriptomic, proteomic, metabolomic, and spatial domains to examine AD as a progressive destabilization of a single integrated disease system organized across multiple interacting biological layers, rather than as a linear cascade. Using an integrative systems framework (Figure 1), we describe how coupled perturbations across proteostatic, metabolic, immune–glial, cytoskeletal, and synaptic modules evolve over time, how buffering capacity progressively erodes toward a non-linear tipping point, and how multi-layer modeling approaches may improve causal inference and therapeutic prioritization compared with single-pathway paradigms.

To align Figure 1 with the 2024 Alzheimer’s Association diagnostic and staging criteria, clinical stage 1 denotes no cognitive or neurobehavioral impairment; stage 2 denotes transitional cognitive decline without MCI-level functional impairment; stage 3 corresponds approximately to MCI; and stages 4–6 correspond to mild, moderate, and severe dementia, respectively. These criteria are not clinical-practice guidelines and do not support routine AD-biomarker evaluation of asymptomatic individuals outside observational or therapeutic research [3].

The proposed network states should therefore be operationalized as candidate research strata. Compensated multi-module stress could require clinical stage 1–2, an abnormal amyloid-pathway (A) Core 1 biomarker, negative tau-PET (T2), absence of MCI-level impairment, and preserved resilience measures—for example, a lower CSF YWHAG ratio and higher resting-state fMRI system segregation. Medial-temporal tau positivity should not be included in this compensated state because amyloid-positive cognitively unimpaired individuals with medial-temporal tau already have substantially greater progression risk than amyloid-positive/tau-negative individuals [27,35,36]. Inflammatory–metabolic amplification could require amyloid positivity plus rising plasma or CSF p-tau217 (T1) or tau-PET extension into medial-temporal or temporal-neocortical regions, together with astrocytic reactivity measured by plasma GFAP and/or AD-pattern FDG-PET hypometabolism. Human studies show that astrocyte reactivity modifies the association of amyloid with phosphorylated tau and subsequent tau-PET accumulation, whereas combined amyloid and neocortical tau are associated with posterior-cingulate hypometabolism and memory decline [37,38]. Blood–brain barrier permeability or CSF soluble PDGFRβ should be recorded separately as a vascular-injury modifier because BBB breakdown has been associated with early cognitive dysfunction independently of amyloid and tau [39]. Decompensated network failure could require high neocortical tau-PET burden, neurodegeneration on structural MRI or FDG-PET, an elevated YWHAG ratio, and reduced network segregation or default-mode-network hub integrity [30,36,40]. These components have not yet been validated jointly and should initially be analyzed as continuous, assay-standardized measures rather than fixed clinical cut-offs.

Clinical action should remain stage-gated. Stages 1–2 support longitudinal observation or enrollment in prevention trials such as AHEAD 3–45, not routine antibody treatment [41]. At stages 3–4, amyloid-directed therapy may be considered where approved after amyloid confirmation and standard safety assessment; the proposed composite state does not itself establish treatment eligibility [16,17]. Its non-core components could instead stratify adjunctive trials—for example, vascular-injury-positive participants for vascular interventions, GFAP/FDG-positive participants for glial or metabolic strategies, and participants with early synaptic or connectomic deterioration for synaptic-network endpoints. Stages 5–6 remain outside the populations for which current anti-amyloid efficacy and safety have been established.

## 2. Multi-Omics Architecture of Alzheimer’s Disease as a Multi-Layer Network Disorder

Alzheimer’s disease is most coherently understood as the progressive destabilization of a single integrated disease system whose behaviour unfolds across multiple, causally linked organizational layers rather than as the accumulation of parallel molecular abnormalities [31,32]. In this framework, different types of omics data (e.g., genomic, transcriptomic, proteomic, and metabolomic) do not provide independent explanations of disease; rather, they capture complementary aspects of disease-related changes at molecular, cellular, and circuit levels. The principal systems-level terms used throughout this framework, along with their operational meanings, are defined in Table 1.

**Figure 2:**
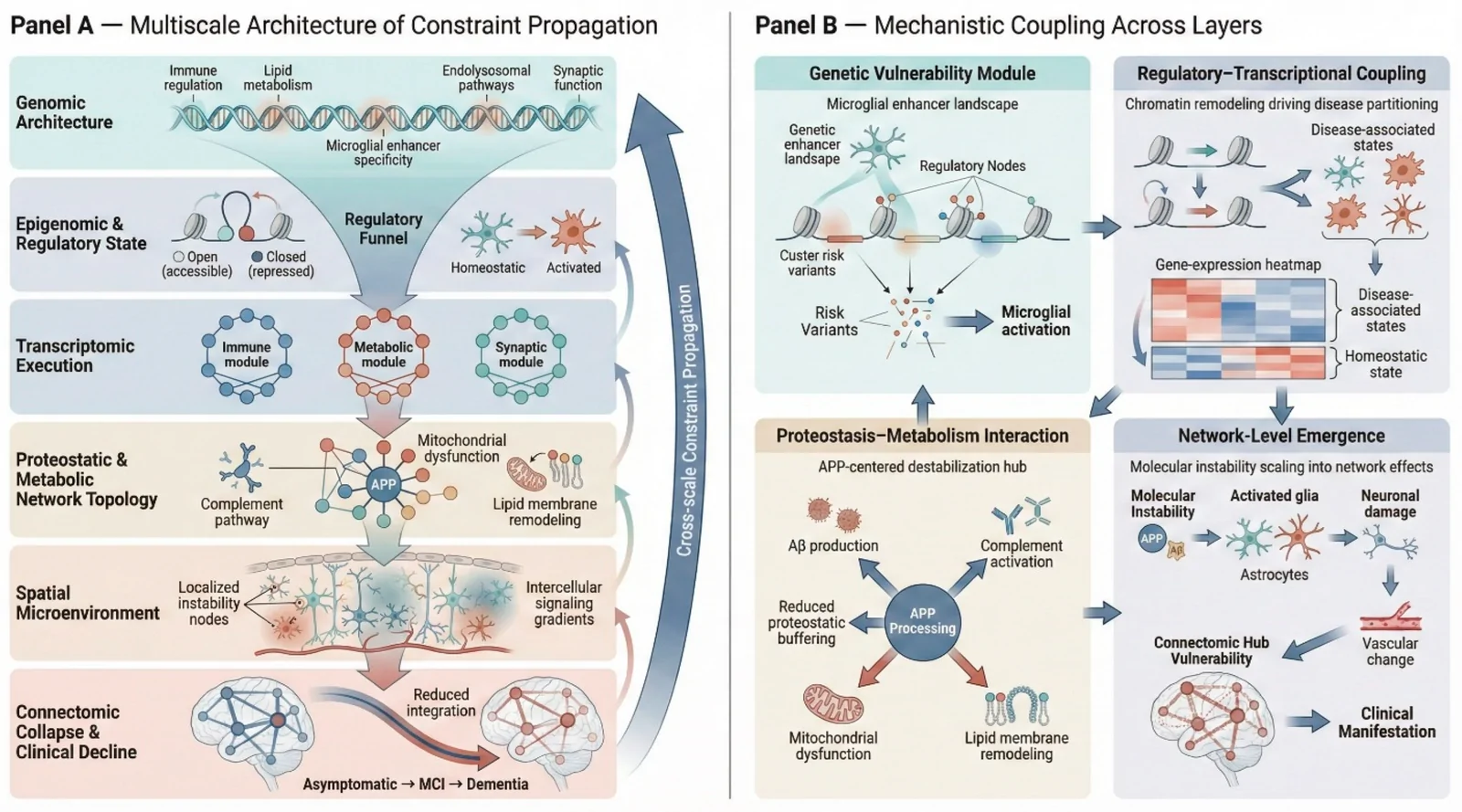
Conceptual model of multiscale constraint propagation in AD. Panel A: Hierarchical architecture. AD is depicted as a progressively coupled multiscale system in which genetic vulnerability constrains regulatory states, partitions transcriptional programmes, and interacts with proteostatic, metabolic, and spatial processes, ultimately scaling to connectomic disruption and clinical decline. The narrowing structure and curved arrow denote proposed cross-scale constraint propagation. Panel B: Cross-layer coupling. Schematic illustration of how genetic risk, regulatory remodeling, and proteostasis–metabolism interactions may amplify from molecular instability to glial activation, neuronal and vascular changes, connectomic hub vulnerability, and clinical manifestation. The framework is integrative and heuristic rather than a definitive causal pathway.

Figure 2 presents a conceptual schematic of this proposed multiscale architecture, offering a heuristic representation of how hierarchical biological layers may become progressively coupled during disease evolution.

Figure 2 can be operationalized as a multilayer graph: Genome-wide association study (GWAS) and quantitative-trait-locus data connect genomic and regulatory layers; ATAC-seq, methylation, and single-cell or single-nucleus RNA-seq define regulatory and cellular states; proteomic, phosphoproteomic, metabolomic, and spatial-omics data define molecular and tissue modules; and diffusion MRI or functional MRI defines structural and functional connectomes. Nodes represent variants, genes, proteins, metabolites, cell states, or brain regions, whereas within-layer and cross-layer edges represent regulatory, molecular, intercellular, and imaging–omics relationships. Longitudinal models can then test whether declining modularity or altered cross-layer coupling precedes clinical transition. For the metabolic layer, orthogonal dual-column gas chromatography–mass spectrometry, mathematical baseline refinement, and chemometric evaluation provide a practical workflow for analytically controlled feature generation before cross-layer integration [42].

The integrated roles of these layers within a unified network model are summarized schematically in Table 2, which serves as an orienting map for the coupled mechanisms described below.

Genomic architecture provides the initial substrate of vulnerability, where inherited variation defines the topology of vulnerability within the network [61]. Large genome-wide association study (GWAS) meta-analyses establish late-onset AD as a polygenic condition in which risk converges on a limited number of stress-handling subsystems, most consistently immune regulation—particularly microglial biology—lipid metabolism, and endolysosomal trafficking [62]. Rather than encoding a deterministic pathogenic sequence, this convergence delineates regions of intrinsically low buffering capacity that are predisposed to failure under cumulative age-related, metabolic, and environmental load [63]. Crucially, most common-risk variants act through non-coding regulatory elements with strong cell-type specificity, embedding fragility directly into regulatory connectivity, especially within microglial enhancer landscapes [48,64]. More broadly, non-coding disease-risk variants are most reliably interpreted when regulatory annotation is resolved by cell state and tissue context and linked to target genes through chromatin, expression, and perturbational evidence [65].

Rare coding variants with larger effect sizes further intensify this inherited fragility by selectively weakening the same buffering subsystems, sharpening the constraints under which downstream adaptive responses must operate [66,67].

Empirically, the largest cited meta-analysis identified 75 loci, including 42 not previously implicated, with prioritized genes enriched in microglial, immune, lipid, and endolysosomal biology [43]. Genomic architecture therefore does not specify a fixed phenotype; it weights cell-type-specific regulatory circuits and narrows the enhancer responses available to the next layer.

Epigenomic regulation can accumulate over long periods, so a cell’s response to a current stress depends partly on its prior regulatory history. Here, path dependence means that earlier DNA-methylation and chromatin-accessibility configurations bias which enhancers can be re-engaged after a later perturbation; therefore, an equivalent present-day insult may elicit different responses in cells with different regulatory histories. The transcriptional state space is the set of gene-expression programmes that a cell can access under defined conditions. Progressive epigenomic remodeling may exclude adaptive states from this set and bias cells toward a narrower repertoire of inflammatory or dysfunctional programmes [47,48].

Consistent with this link, a multiregion atlas integrating 3.5 million cells from 384 postmortem brain samples mapped more than 1 million candidate cis-regulatory elements across 67 cell subtypes [47]. Cell-type-specific enhancer architecture therefore restricts the transcriptional programmes that can be recruited under stress, providing a measurable regulatory-to-transcriptomic constraint.

Epitranscriptomic regulation links transcriptional output to protein synthesis by controlling RNA stability and translation without altering DNA sequence or chromatin state. Multiregion human-brain mapping demonstrates spatial heterogeneity of N6-methyladenosine (m6A) and enrichment near neurodegeneration-associated loci [68]. In affected AD cortex and experimental models, methyltransferase-like 3 (METTL3) expression and global m6A abundance are increased in specific regions [69]. Neuronal METTL3 increases m6A deposition on leucine-rich repeat and immunoglobulin-like domain-containing protein 2 (LINGO2) mRNA. This promotes YTH N6-methyladenosine RNA-binding protein 2 (YTHDF2)-mediated decay, lowers LINGO2 abundance, facilitates amyloid precursor protein (APP)–β-site APP-cleaving enzyme 1 (BACE1) interaction, and increases amyloid-β production [69]. Conversely, genetic or pharmacological reduction of METTL3 mitigates amyloid pathology and cognitive deficits in AD models [57]. Separate human cortical mapping identifies altered m6A distribution in transcripts involved in synaptic function and RNA metabolism [70]. Activated microglia also show increased METTL3 activity in manganese- and α-synuclein-induced neuroinflammatory models and in postmortem AD, Parkinson’s disease, and dementia with Lewy bodies tissue [59]. METTL3-dependent modification of interleukin-1β and interleukin-6 transcripts engages YTHDF2 and insulin-like growth factor 2 mRNA-binding protein readers, thereby linking RNA modification to inflammatory output [71].

Transcriptomics therefore reflects not isolated gene dysregulation but the execution of programmes permitted by upstream genetic and epigenomic constraints. Systems-level analyses consistently demonstrate coordinated module-level rewiring across neuronal and glial populations, while single-cell studies resolve these patterns into structured cell-state transitions, including disease-associated microglial phenotypes, selectively vulnerable neuronal subtypes, and astrocytic programmes linked to either degeneration or resilience [47]. These transcriptional states emerge because the regulatory system can no longer explore the full repertoire of adaptive responses, leading to progressively stereotyped patterns of programme execution that differ by region and cellular context.

Same-individual integration of up to 1358 aged human brain samples resolved 11 molecular subtypes, including three AD-associated clusters with distinct immune, metabolic, and synaptic configurations [50]. These permitted transcriptional states determine the synthesis and turnover demands transmitted to the proteomic layer, while post-transcriptional regulation explains why RNA and protein changes are not fully concordant.

Proteomic and phosphoproteomic studies in AD show that protein-level alterations are only partially concordant with corresponding RNA changes [72]. In addition, phosphorylation changes represent a distinct regulatory layer not captured by transcriptomic data alone. Accordingly, transcriptomic measurements do not fully predict protein abundance or protein-state changes [72]. This transition is tightly linked to declining proteostasis capacity, which acts as a cross-layer constraint coupling metabolism, lipid trafficking, and protein turnover. Age-related impairment of proteasomal and autophagic pathways synchronises failure across otherwise distinct molecular modules, rendering network collapse increasingly irreversible once proteostatic buffering is exhausted [73].

Quantitative proteomics across more than 2000 human brains and nearly 400 cerebrospinal-fluid samples identified an early sugar- and energy-metabolism module enriched for microglial and astrocytic proteins [74]. Protein abundance and phosphorylation states therefore set the burden on folding, degradation, signaling, and energy systems, constraining metabolic feasibility at the next layer.

Metabolomics and lipidomics further constrain this system by defining its energetic and structural feasibility. Bioenergetic capacity, redox balance, and substrate availability are closely linked to multiple cellular processes, including proteostasis, synaptic transmission, and immune regulation [75]. Declining metabolic reserve reduces tolerance to perturbation regardless of regulatory or transcriptional intent, accelerating network destabilization as energetic thresholds are approached [76]. Lipid organization imposes an additional structural constraint by shaping membrane domains, endosomal trafficking, and proteolytic localization, thereby modulating signal compartmentalization and protein turnover. Genetic and mechanistic convergence on lipid-handling pathways underscores that membrane organization is not a downstream consequence but a rate-limiting condition that conditions how dysfunction propagates across cellular networks [77].

Experimental AlCl_3_ toxicology provides a complementary cross-compartment example in which oxidative, mitochondrial, and inflammatory effects extend beyond the brain; such models can test central–peripheral coupling but should not be interpreted as evidence that aluminum causes sporadic AD [78].

At the genetic–metabolic interface, analysis of early-onset AD (n = 19,668) and lipid traits (n = 1,320,016) identified five shared loci and three regions of local genetic covariance [54]. The resulting energetic and membrane constraints are filtered by perfusion, cell composition, and intercellular exchange, thereby determining which tissue neighborhoods enter a vulnerable spatial state.

These molecular and metabolic constraints are embedded within spatially structured tissue environments, which determine where network failure first becomes biologically consequential. Spatial and single-cell multi-omics reveal that instability emerges at the tissue and cellular level within specific neighborhoods, cortical layers, and regions where vulnerability, metabolic stress, and intercellular signaling intersect [47,55]. Local interactions among neurons, glia, vasculature, and extracellular milieu can transiently stabilize or amplify dysfunction, creating focal points from which failure propagates. Neurovascular coupling plays a critical integrative role at this stage, linking metabolic insufficiency and inflammatory signalling to both local cellular environments and large-scale network integrity [9].

Cross-omic integration also resolved four multimodal molecular profiles with distinct cognitive decline, neurodegeneration, astrogliosis, and survival [55], demonstrating that comparable pathology can occupy different tissue-level states. Recurrent failure in high-demand or poorly perfused neighborhoods can then preferentially destabilize synapses and high-centrality nodes, transmitting spatial constraints to the connectomic layer.

Within the proposed multiscale architecture, the neurovascular unit (NVU) functions not as a passive casualty of neurodegeneration but as an active mesoscale constraint interface. Human single-nucleus and spatially informed transcriptomic studies identify inflammatory endothelial state reprogramming in AD, including NF-κB–associated modules enriched for junctional remodeling and endothelial-to-mesenchymal transition signatures that correlate inversely with cognition [79,80]. Convergent imaging and biomarker data further demonstrate that blood–brain barrier dysfunction emerges early and predicts cognitive decline, reframing vascular instability as a temporal inflection point rather than a late epiphenomenon [81]. Mechanistically, multi-omics analyses in APOE4 models show that endothelial and pericyte signaling dysregulation—affecting junctional integrity, cytoskeletal organization, and transcytosis—precedes synaptic interactome disruption and behavioral deficits, positioning vascular transcriptomic collapse upstream of circuit failure [82]. Disrupted endothelial–pericyte crosstalk impairs neurovascular coupling, capillary perfusion, and amyloid clearance pathways, creating a feed-forward loop between metabolic insufficiency, inflammation, and proteostatic stress [83,84].

Within Figure 2, the neurovascular unit is positioned between metabolic/lipid constraints and connectomic vulnerability. Early endothelial and pericyte dysfunction reduces substrate delivery and clearance, while microglial cytokines and astrocytic end-foot or aquaporin-4 alterations weaken barrier integrity and neurovascular coupling; the resulting hypoperfusion and leakage further intensify microglial activation, astrocyte reactivity, and synaptic energy failure [9,37,81,84,85,86,87]. Over time, this bidirectional loop converts local immune–metabolic stress into spatially patterned synaptic loss, particularly in energetically costly hub regions. Vascular instability can therefore precede and subsequently amplify circuit failure without displacing the earlier immune, lipid, or endolysosomal mechanisms.

At the macroscopic scale, connectomics captures the cumulative outcome of this multiscale constraint propagation. Structural and functional connectivity studies reveal progressive loss of hub integrity, reduced efficiency, and impaired integration across distributed cognitive networks [88]. Within an integrated framework, these connectomic alterations are emergent consequences of accumulated genetic, regulatory, metabolic, proteostatic, and spatial constraints acting on circuit-level topology, rather than primary drivers. Once large-scale network integration is compromised, information flow degrades and cognitive function declines, closing the causal loop between molecular vulnerability and clinical manifestation [89].

Consistent with this final link, longitudinal and dynamic functional-connectivity studies report stage-dependent brain-state disruption and default-mode-network change [58,60], while frontoparietal control-network connectivity moderated amyloid-associated cognitive decline in the A4 cohort (interaction *p* = 0.025) [59]. Connectomic integrity therefore constrains clinical expression: similar molecular burdens can yield different cognitive outcomes depending on residual hub and edge redundancy.

Taken together, the multi-omics architecture of AD describes a single, temporally evolving failure process in which inherited vulnerability constrains regulatory flexibility, regulatory constraint shapes transcriptional execution, execution stresses proteostatic and metabolic capacity, energetic and lipid limitations erode feasibility, local failures propagate through tissue architecture, and large-scale network integration ultimately collapses. The disease therefore emerges not from parallel pathological pathways but from a progressive loss of coordination across interacting network layers, each inheriting and amplifying constraints imposed by the upstream levels.

Within this framework, the dominant causal direction is conceptualized as cross-scale constraint propagation, in which inherited and regulatory-level factors progressively shape transcriptional, proteostatic, metabolic, spatial, and connectomic states. However, this hierarchy is not strictly unidirectional. Feedback interactions are also evident, including glial-state remodeling of regulatory landscapes, metabolic and inflammatory influences on cellular state transitions, and circuit-level dysfunction that further amplifies local molecular and cellular stress, indicating that AD progression reflects a dynamically coupled system rather than a purely linear cascade.

These concepts can be tested using three prespecified quantities. Within each omics or connectomic layer, module redundancy would be quantified as the proportion of node pairs in a functional module connected by at least two independent paths; a lower proportion indicates reduced buffering [90]. For adjacent layers (X) and (Y), constraint propagation is supported when (Xt) predicts (Y{t + 1}) after adjustment for (Yt), relevant covariates, and the reverse path; the standardized lagged coefficient provides the cross-layer coupling estimate. This can be fitted using a cross-lagged multilevel or dynamic Bayesian model, with quantitative-trait loci or experimental perturbations used to orient edges [31]. In longitudinal cohorts, the resilience curve is the within-person trajectory of synaptic, connectivity, or cognitive function against cumulative amyloid, tau, and neurodegeneration burden. Buffering loss is indicated by a progressively steeper negative slope, tested by a pathology-by-time interaction, while a piecewise mixed-effects model can estimate the breakpoint at which decline accelerates [91,92].

## 3. Mechanistic Network Modules Underlying Alzheimer’s Disease

Alzheimer’s disease can be mechanistically reframed as failure within a single integrated disease system, expressed through the progressive coupling and destabilization of interacting functional modules rather than a sequential, single-cascade disorder. In this framework, “modules” are not isolated pathways but semi-autonomous subgraphs—densely interconnected processes with characteristic feedback loops, that are temporarily buffered from one another. Disease progression reflects progressive depletion of this buffering capacity and strengthening of cross-module coupling, such that localized stress increasingly propagates across the system. The subsections below describe a small number of high-order modules that are repeatedly recovered across human genetics, multi-omics integration, cell-state atlases, and connectome-informed propagation models, and whose interaction structure best explains nonlinear progression and threshold-dependent clinical emergence.

Temporally, these modules appear to become coupled in a staged but overlapping manner rather than as independent processes. In early disease, glial responses may act as modulatory factors that condition the relationship between amyloid burden and downstream tau pathology [37]. During Prodromal/MCI due to AD (clinical stage 3), proteostatic and metabolic disturbances become increasingly interdependent, while experimental studies indicate that reducing APP expression can restore endolysosomal and autophagic function and reduce amyloid aggregation, suggesting that specific intracellular pathways remain modifiable at Preclinical AD (clinical stages 1–2) [93]. In early symptomatic stages, synaptic dysfunction becomes a major determinant of clinical expression, consistent with biomarker studies showing that synaptic markers explain substantial variance in cognitive decline beyond amyloid and tau [36].

This temporal coupling also suggests that therapeutic reversibility is stage-dependent. Clinical trials targeting amyloid in early symptomatic AD (MCI or mild dementia; clinical stages 3–4) demonstrate modest slowing of cognitive decline, with greater effects observed in individuals with lower tau burden, indicating that earlier stages remain more amenable to modifying disease trajectories [14,15]. In contrast, later stages dominated by synaptic and network-level dysfunction are more consistent with strategies aimed at preserving residual function and slowing further decline.

### 3.1. Proteostasis, Trafficking, and Protein Stress Amplification

Within a systems-level framework of AD, convergent human genetic, transcriptomic, and cellular evidence position endolysosomal pathway vulnerability within the molecular layer as a central and early pathogenic substrate. Large-scale meta-analysis of human CNS transcriptomes demonstrates coordinated downregulation of lysosomal acidification machinery, ubiquitin–proteasome components, and mitochondrial oxidative phosphorylation across neurodegenerative disorders including AD [10]. Region-specific cortical profiling confirms suppression of neuronal proteostasis modules [94], while selectively vulnerable precuneus neurons exhibit early dysregulation of endosomal–lysosomal and autophagy-related genes [95]. Pathway-specific polygenic risk mapping further demonstrates that AD risk variants enriched in endolysosomal network genes correlate directly with enlarged neuronal endosomes and microglial lysosomes in human brain tissue, independent of plaque burden [52], establishing organelle-level dysfunction as a genetically anchored vulnerability.

Within this destabilized degradative landscape, lysosomal membrane integrity and acidification capacity emerge as critical control elements. Aging-derived human neurons exhibit constitutive lysosomal membrane damage and impaired repair capacity [96], and disruption of lysosome quality control mechanisms—including membrane repair and lysophagy—has been identified as a mechanistic amplifier of neurodegenerative stress [97]. In this context, APP processing becomes highly sensitive to vesicular routing. Cholesterol retention within endosomal–lysosomal compartments increases APP C-terminal fragments and Aβ without altering transcription, demonstrating that altered trafficking topology alone can bias amyloidogenic flux [98].

Recent mechanistic advances further reposition APP carboxy-terminal fragments (APP-CTFs), particularly C99, as proximal destabilizers of endolysosomal dynamics. APP-CTFs impair lysosomal function and perturb ER–lysosome calcium signaling independently of Aβ [11], while direct interaction between C99 and Rab5 promotes endosomal enlargement and trafficking abnormalities [99]. Complementarily, modest elevations of endogenous APP-βCTF increase lysosomal pH and reduce cathepsin activity, constraining degradative flux [100]. Endolysosomal dysfunction extends beyond neurons: loss of the AD risk gene SORL1 disrupts lysosomal hydrolase trafficking in human microglia-like cells, reducing degradative efficiency despite increased amyloid uptake [101].

Superimposed on trafficking-dependent destabilization, metabolic and ER stress amplify amyloidogenic capacity through translational control. Energy deprivation induces eIF2α phosphorylation, increasing BACE1 translation and Aβ production [102], and PERK reduction attenuates BACE1 elevation and amyloid burden in vivo [103]. Elevated eIF2α phosphorylation in AD brains [104] links metabolic strain to amyloidogenic amplification, while incomplete suppression of BACE1 following eIF2α inhibition indicates convergence of translational and trafficking mechanisms [105]. Reducing APP dosage via antisense oligonucleotides restores endolysosomal and autophagic function in human cortical neurons and reduces amyloid aggregation [93], providing causal support for this integrated model.

**Figure 3:**
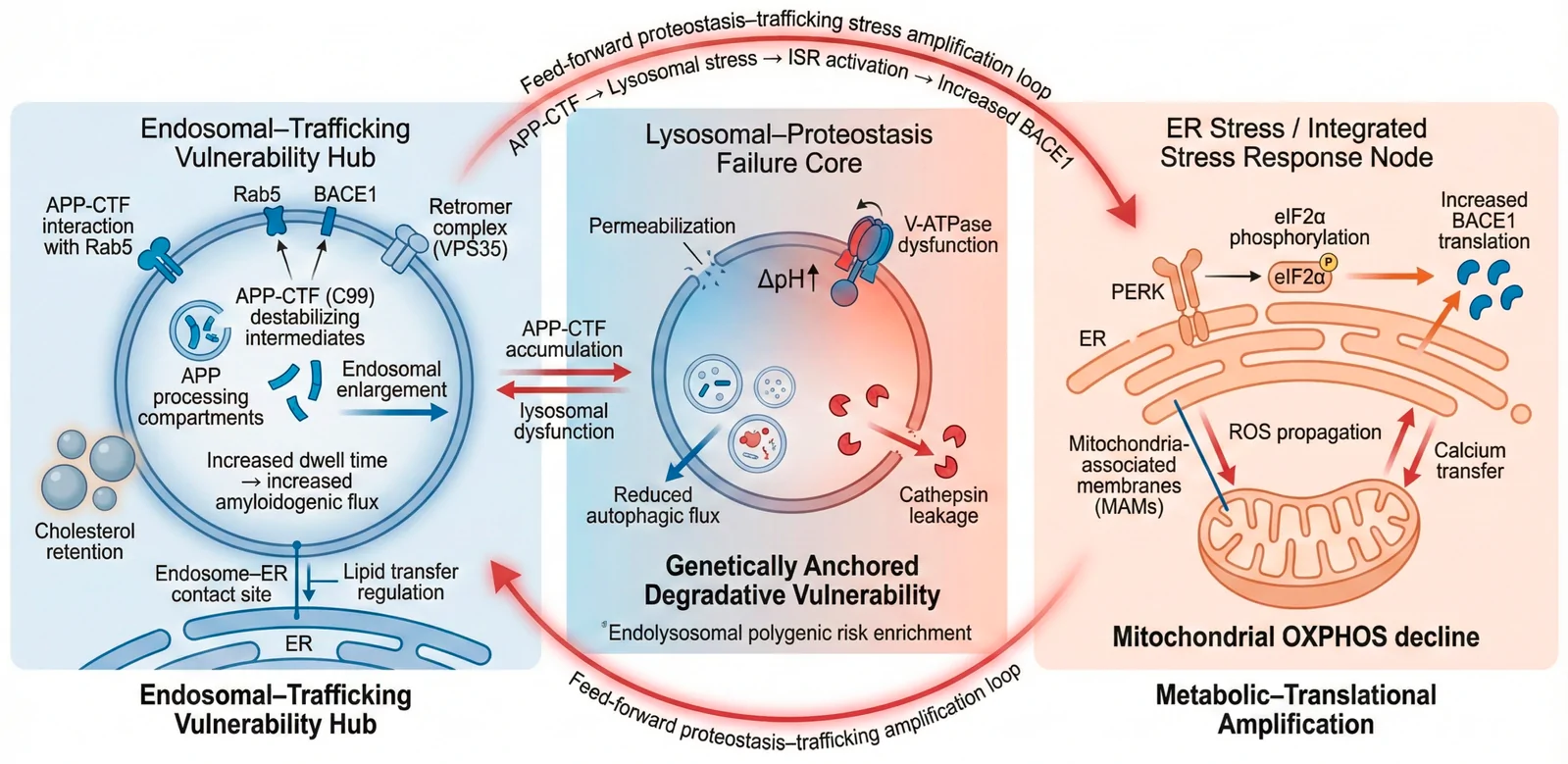
Systems-level conceptual model of genetically anchored endolysosomal dysfunction linking lysosomal acidification failure, APP-CTF accumulation, and ISR-driven BACE1 upregulation to proteostatic stress amplification in AD.

As depicted in Figure 3, these converging mechanisms form a feed-forward proteostasis–trafficking stress amplification network in which genetically anchored lysosomal vulnerability, APP-CTF–mediated endosomal expansion, impaired acidification, microglial degradative deficits, and ISR-driven translational upregulation converge through organelle contact-site coupling to convert localized stress into system-wide destabilization. In this configuration, amyloidogenic processing functions not as an isolated initiating cascade but as a stress-amplifying output of a progressively compromised intracellular degradation network.

In summary, convergent genetic, transcriptomic, and cellular evidence indicates that endolysosomal dysfunction constitutes an early, genetically anchored vulnerability in AD, in which impaired proteostasis, disrupted trafficking, and stress-responsive translational control converge to amplify amyloidogenic processing. Collectively, these coupled mechanisms establish a feed-forward proteostasis–trafficking stress network that transforms localized organelle dysfunction into system-level instability.

### 3.2. Cytoskeletal Transport, Tau Propagation, and Network Vulnerability

As proteostatic buffering declines, cytoskeletal regulation becomes increasingly sensitive to post-translational perturbations of tau. Beyond classical aggregation models, tau pathology is now understood to involve liquid–liquid phase separation (LLPS) as an early, regulated intermediate. Tau undergoes LLPS through electrostatic interactions between oppositely charged domains, forming condensates whose material properties determine their subsequent fate [12,106]. Phosphorylation patterning critically modulates this process: phosphomimetic substitutions that enhance charge polarization increase LLPS propensity and alter droplet maturation kinetics [107]. Distinct phosphosite clusters differentially influence condensate stability and microtubule-binding affinity, indicating that kinase-network imbalance alters both phase behavior and cytoskeletal engagement.

Disease-relevant cofactors further tune condensate dynamics. Zinc binding promotes LLPS and aggregation by engaging high-affinity sites within the repeat domain [108], while RNA G-quadruplexes and Ca^2+^ synergistically accelerate liquid-to-gel transition under physiological ionic conditions [109]. Single-molecule FRET analyses demonstrate that tau transitions from compact to extended conformations during LLPS, directly linking monomer structure to condensate assembly [110]. Importantly, condensate maturation is context-dependent rather than inevitable: heterotypic chaperone-mediated phase separation, such as Ydj1–tau condensates, suppresses fibrillization [111], whereas TRIAD3A-mediated condensates accelerate fibril formation within droplets [112]. Thus, tau condensation represents a tunable kinetic checkpoint whose material state influences aggregation probability.

Tau liquid–liquid phase separation may also intersect with extracellular release, although the direct causal evidence remains incomplete. Tau can exit cells through phosphatidylinositol-4,5-bisphosphate-dependent translocation across the plasma membrane and through vesicular or lysosomal routes; phosphorylation, oligomerization, cell-surface heparan-sulfate proteoglycans, and membrane composition influence this export [113,114]. Separately, anionic membrane surfaces concentrate full-length tau and can induce a macroscopic condensed or gel-like phase together with β-sheet-rich oligomerization [115]. These findings support a model in which membrane-associated condensation raises the local tau concentration and may facilitate direct translocation, vesicular loading, or release of seed-competent species into the extracellular space. However, selective disruption of tau phase separation has not yet been shown to reduce extracellular tau independently of membrane binding, oligomerization, or aggregation. The LLPS–secretion connection should therefore be treated as a testable coupling mechanism rather than an established secretory pathway.

At the cytoskeletal interface, hyperphosphorylation disrupts cooperative tau binding to microtubules, impairing envelope formation and altering motor accessibility [116]. Tau differentially modulates motor isoforms: KIF1A processivity is strongly reduced under load, whereas KIF5C inhibition is milder, and dynein team force generation shifts depending on motor composition [116,117]. In tauopathy models, anterograde mitochondrial transport declines while retrograde flux remains comparatively preserved, reflecting reduced kinesin engagement and sustained dynein activity [118]. Direct tau–kinesin interaction further inhibits kinesin ATPase activity and influences tau stability and autophagic turnover [119]. Together, these data indicate that tau pathology induces cargo- and isoform-specific transport friction rather than uniform axonal collapse.

The energetic consequences scale with projection length. Reduced distal mitochondrial delivery shifts ATP gradients and amplifies oxidative stress in synaptic compartments. Mitochondrial multi-omics analyses confirm coordinated alterations in respiratory chain components, calcium handling, and redox pathways in AD brain [120], supporting tight coupling between transport impairment and metabolic stress.

Importantly, multi-omics integration demonstrates that this cytoskeletal–energetic vulnerability is embedded within broader cross-system reorganization. Same-individual brain multi-omics integration identifies AD-associated factors characterized by immune epigenetic activation, reduced heat-shock proteostasis programmes, disrupted cytoskeletal proteomics, and impaired energy metabolism [50]. Tau PET–guided proteomic–transcriptomic integration further reveals metabolic–cytoskeletal modules whose regional expression correlates with tau burden and cognitive decline [121]. Integrative modelling shows that functional connectivity predicts tau presence across regions, whereas regional gene-expression gradients modulate accumulation magnitude [122]. Thus, neurons with long projection length and high graph centrality accumulate transport load and metabolic cost disproportionately.

**Figure 4:**
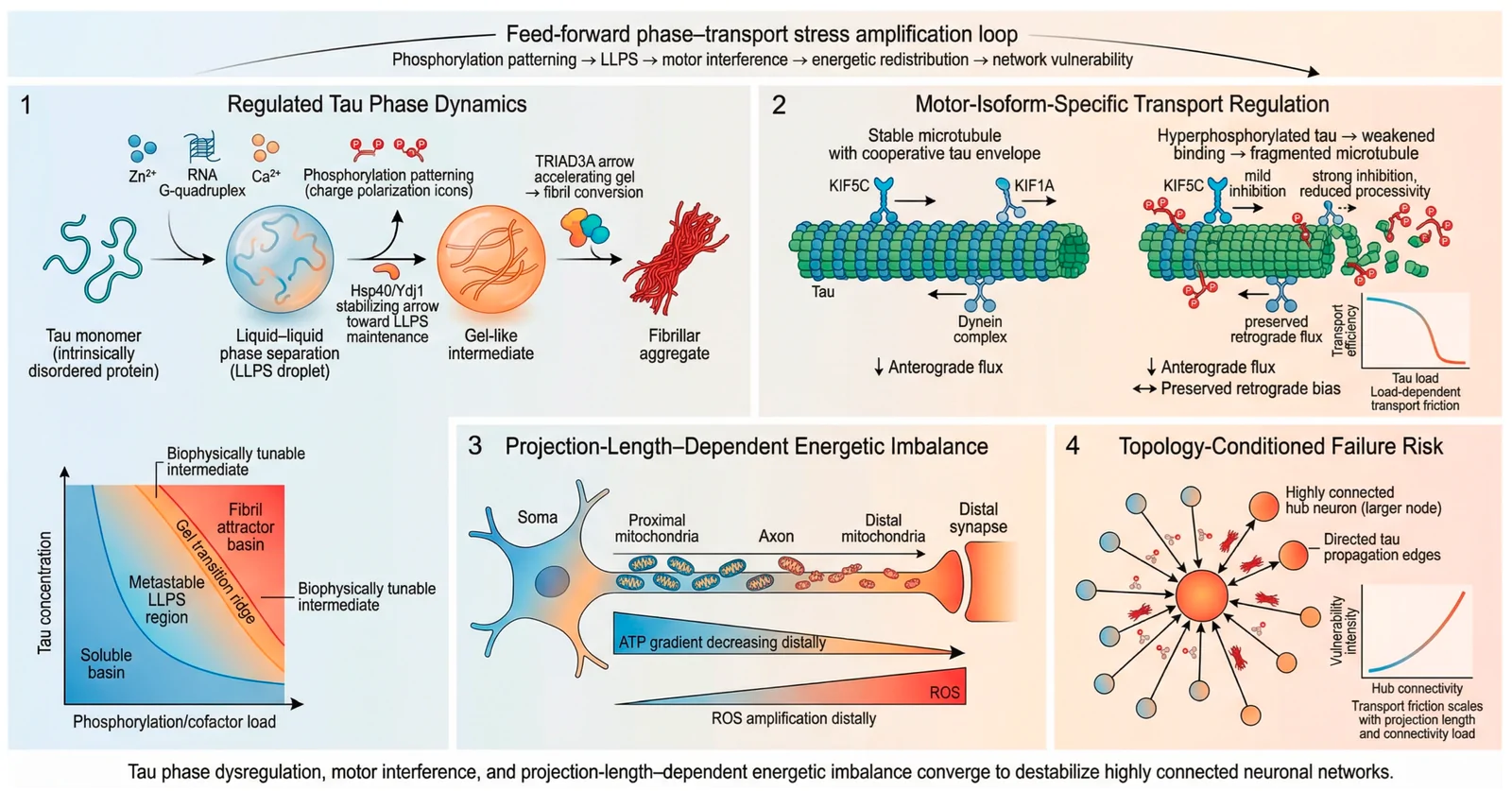
Phase-transition–coupled axonal transport destabilization and topology-conditioned vulnerability in AD. (**1**) Phosphorylation-dependent tau phase transitions regulate liquid–liquid phase separation (LLPS), condensate maturation, and fibrillization. (**2**) Hyperphosphorylated tau disrupts cooperative microtubule binding and differentially impairs motor isoforms, producing reduced anterograde transport and transport friction. (**3**) Projection-length–dependent mitochondrial redistribution lowers distal ATP and increases oxidative stress. (**4**) Highly connected hub neurons accumulate disproportionate transport load, creating topology-conditioned vulnerability and amplified network failure.

As illustrated in Figure 4, phosphorylation-dependent phase transitions, motor-isoform–specific transport interference, and projection-length–dependent energetic imbalance converge within a topology-conditioned vulnerability landscape.

Tau therefore functions not simply as an aggregating protein but as a phase-transition–coupled transport destabilizer whose molecular state interacts with multi-omic network reorganization to amplify failure risk in highly connected neuronal systems.

In summary, tau pathology acts as a phase-transition–dependent regulator of cytoskeletal transport, in which LLPS dynamics, phosphorylation-dependent microtubule disengagement, and motor-specific transport disruption collectively impair axonal trafficking and energy distribution. Collectively, these coupled processes embed tau-mediated dysfunction within a topology-dependent vulnerability landscape, linking molecular state transitions to system-level network instability.

### 3.3. Synaptic Integrity, Glial Reprogramming, and Edge Failure

The convergence of proteostasis stress and cytoskeletal transport impairment is most directly expressed at synapses, which constitute the physical edges of neuronal networks and the primary substrate of cognitive computation. Quantitative postmortem studies consistently demonstrate that synapse number correlates strongly with cognitive performance and often shows tighter associations with Mini-Mental State Examination scores than plaque burden. Stereological analyses in inferior temporal and posterior cingulate cortex confirm significant synaptic loss across prodromal and clinical AD stages, with synapse density closely tracking cognitive decline [123,124]. A meta-analysis of 417 studies further identifies consistent vulnerability of presynaptic vesicle and synaptic machinery proteins in AD [125]. These findings support a network interpretation in which cognitive failure emerges when synaptic connections at the cellular and circuit level are progressively removed or destabilized.

Recent large-scale cerebrospinal fluid proteomic analyses extend this network-based interpretation into living human cohorts. Across 3397 individuals, the synaptic protein ratio YWHAG:NPTX2 was shown to index a maladaptive synaptic network state—reflecting loss of homeostatic synaptic regulation rather than synapse number alone—that explained cognitive impairment beyond amyloid-β, tau PET, and established neurodegeneration markers, and predicted long-term cognitive trajectories independent of classical A/T/N status [36]. This demonstrates that synaptic protein network state constitutes an independent determinant of cognitive trajectory, consistent with a model in which edge integrity rather than aggregate pathology load governs network failure.

Consistent with this framework, tau PET–anchored multi-omics network analysis demonstrated that elevated regional tau PET signal and cognitive decline coincide with coordinated dysregulation of bioenergetic metabolism, cytoskeletal integrity, synaptic regulation, and mTOR- and AMPK-linked nutrient-sensing pathways, indicating that tau pathology emerges within a broader failure of cellular maintenance and signaling processes within the disease system rather than as an isolated protein aggregation process [121].

**Figure 5:**
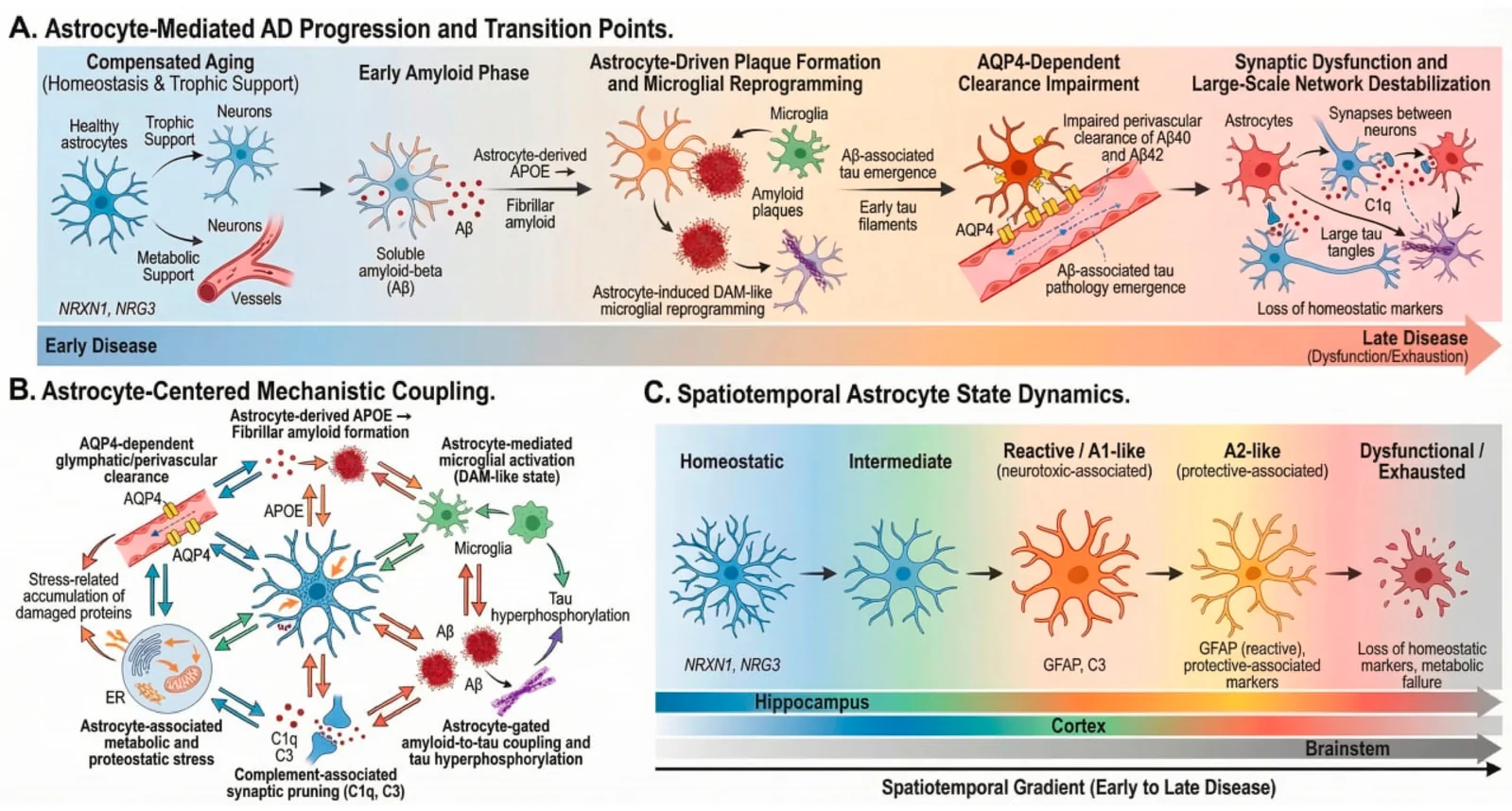
Astrocyte-centered multi-layer framework of Alzheimer’s disease progression. (**A**) Astrocytes regulate key transition points linking amyloid accumulation to tau pathology, microglial reprogramming, and network destabilization. APOE-driven fibrillar Aβ formation and AQP4-dependent clearance shape progression from compensated states to synaptic dysfunction. (**B**) Astrocytes integrate lipid, immune, synaptic, and proteostatic pathways within a coupled system, including amyloid aggregation, microglial activation, complement-mediated pruning, and amyloid-to-tau coupling. (**C**) Astrocytes exhibit dynamic, stage- and region-dependent transitions from homeostatic to reactive and dysfunctional states, reflecting context-dependent reprogramming that contributes to disease progression.

Within this network framework, astrocytes emerge as central components, as recent evidence demonstrates their direct involvement in multiple quantitatively defined transitions in AD progression (Figure 5).

At the level of disease transitions, Bellaver et al. analyzed 1016 cognitively unimpaired individuals and showed that amyloid burden was associated with increased plasma p-tau181 only in astrocyte-reactive individuals (β = 0.34 vs. 0.04; interaction *p* < 0.0001), thereby linking astrocyte reactivity to the transition from amyloid accumulation to tau pathology [37], as demonstrated in the progression framework shown in Figure 5A. Consistent with this transition-level role, Manescu et al. further demonstrated that astrocyte-mediated clearance mechanisms directly influence amyloid burden [85], as AQP4 facilitation reduced Aβ levels and improved behavioral outcomes, whereas inhibition increased both Aβ40 and Aβ42, reinforcing the role of astrocytes in shaping key disease inflection points (Figure 5A).

These transition-level effects are underpinned by coordinated astrocyte-centered mechanisms that couple multiple pathological axes within a unified system (Figure 5B). In this context, Preman et al. showed that astrocyte-derived APOE is sufficient to restore fibrillar plaque formation and induce DAM-like microglial reprogramming, with APOE4 exhibiting a dose-dependent association with Aβ42 (R^2^ = 0.61, *p* = 0.007), thereby linking astrocytic lipid metabolism to both amyloid deposition and immune activation [86]. In parallel, astrocyte-mediated complement signaling contributes to synaptic remodeling, indicating that inflammatory, metabolic, and synaptic processes are not independent but functionally coupled through astrocyte-mediated interactions (Figure 5B).

At the cellular level, these coupled mechanisms manifest as dynamic and heterogeneous astrocyte states that evolve across disease progression (Figure 5C). Accordingly, Serrano-Pozo et al. conducted single-nucleus transcriptomic profiling of 628,943 astrocytes across five brain regions from 32 donors [87], revealing structured, stage- and region-dependent astrocyte states, including a progressive loss of trophic-supporting subpopulations and late-stage exhaustion patterns [87]. Extending this, Kim et al. described diverse pathological phenotypes, including A1-like neurotoxic and A2-like protective states, alongside apoptotic, ferroptotic, and senescent profiles [126], indicating broad functional reprogramming across inflammatory, metabolic, and synaptic domains (Figure 5C). Taken together, these interconnected observations position astrocytes as mesoscale regulators that couple molecular, cellular, and synaptic processes across biological layers, including amyloid deposition, tau emergence, microglial state transitions, synaptic remodeling, and clearance failure within the multi-layer network architecture of AD.

Single-cell and spatially resolved analyses indicate that synaptic vulnerability is gated by circuit activity states, with hyperactive synapses preferentially acquiring complement tags that bias them toward elimination within an interferon-primed microglial milieu rather than through baseline homeostatic pruning [127]. Importantly, this immune gating is not spatially diffuse. High-plex spatial proteomic mapping of human Alzheimer’s disease cortex demonstrates that neuroimmune activation is organized into anatomically restricted microenvironments rather than diffusely distributed inflammation. Imaging mass cytometry identifies plaque-proximal microglial clusters enriched for complement components, interferon-responsive proteins, and APOE–TREM2–associated lipid programmes that are spatially confined to micrometer-scale domains, while adjacent glial populations maintain distinct molecular phenotypes. These findings indicate that inflammatory activation in Alzheimer’s disease is spatially compartmentalized within lesion-centered tissue neighborhoods. Such observations align with broader multi-omics syntheses emphasizing the value of high-spatial-resolution approaches for resolving localized cellular states in neurodegenerative disorders [128]. Together, these observations support a model in which immune-mediated synaptic injury is locally organized within discrete cortical niches, such that edge removal occurs in spatially bounded microenvironments whose impact on cognition is determined by the topological embedding of the affected synapses rather than by diffuse inflammatory burden.

At a mechanistic level, failure of microglial cytosolic DNA clearance initiates a sustained innate immune state rather than a transient inflammatory response. DNase II deficiency leads to lysosomal dysfunction and accumulation of endogenous double-stranded DNA within the cytoplasm, which constitutively activates the cGAS–STING pathway and downstream TBK1–IRF3 signaling. This results in persistent type I interferon production and STAT1-dependent transcriptional reprogramming, shifting microglia away from homeostatic surveillance toward a disease-associated immune phenotype. Within this state, interferon signaling induces complement gene expression and enhances phagocytic capacity, thereby coupling intrinsic nucleic acid stress to aberrant synaptic engulfment. The same immune milieu further amplifies amyloidogenic processing and tau phosphorylation, establishing a feed-forward loop linking microglial immune dysregulation, synaptic destabilization, and proteinopathy [129]. This mechanism positions microglial DNA sensing as an upstream integrator that converts intracellular stress signals into maladaptive circuit remodeling, rather than a secondary response to neuronal degeneration.

Cell-resolved and transcriptomic analyses converge on a model in which synaptic failure reflects state-dependent neuronal destabilization rather than variation in amyloid burden or gliosis magnitude. Integration of single-nucleus transcriptomics, synaptic imaging, and cortical profiling demonstrates coordinated downregulation of SNARE-associated and synaptic transmission genes in excitatory and inhibitory neurons, preferentially affecting highly connected synaptic network hubs. Perturbation of neuronal surface signaling alone is sufficient to induce synapse loss and tau phosphorylation while leaving plaque burden and glial activation largely unchanged, directly linking disruption of neuronal synaptic connectivity to cognitive decline in AD [130,131].

Same-individual multi-omics factor analysis identifies Alzheimer’s-associated latent programmes in which increased H3K9ac enrichment at immune and interferon-related loci coincides with reduced transcription of heat-shock and protein-folding genes, alongside proteomic disruption of cytoskeletal organization, mitochondrial matrix components, and ubiquitination-linked protein systems [50]. These convergent alterations are captured within a single molecular factor that tracks tau pathology and cognitive decline, indicating that synaptic degeneration arises in the context of coordinated failure of cellular maintenance, energy metabolism, and structural integrity rather than from synapse-restricted pathology.

At the macroscopic scale, connectome structure constrains where tau pathology appears, whereas regional vulnerability determines the extent of tau accumulation. Multi-scale modeling shows that tau presence follows connectivity-mediated propagation at the macroscopic circuit level, while local tau burden is shaped by intrinsic factors such as MAPT expression, amyloid burden, cerebral perfusion, and excitatory–inhibitory balance, thereby separating mechanisms of spread from mechanisms of amplification in AD [122].

**Figure 6:**
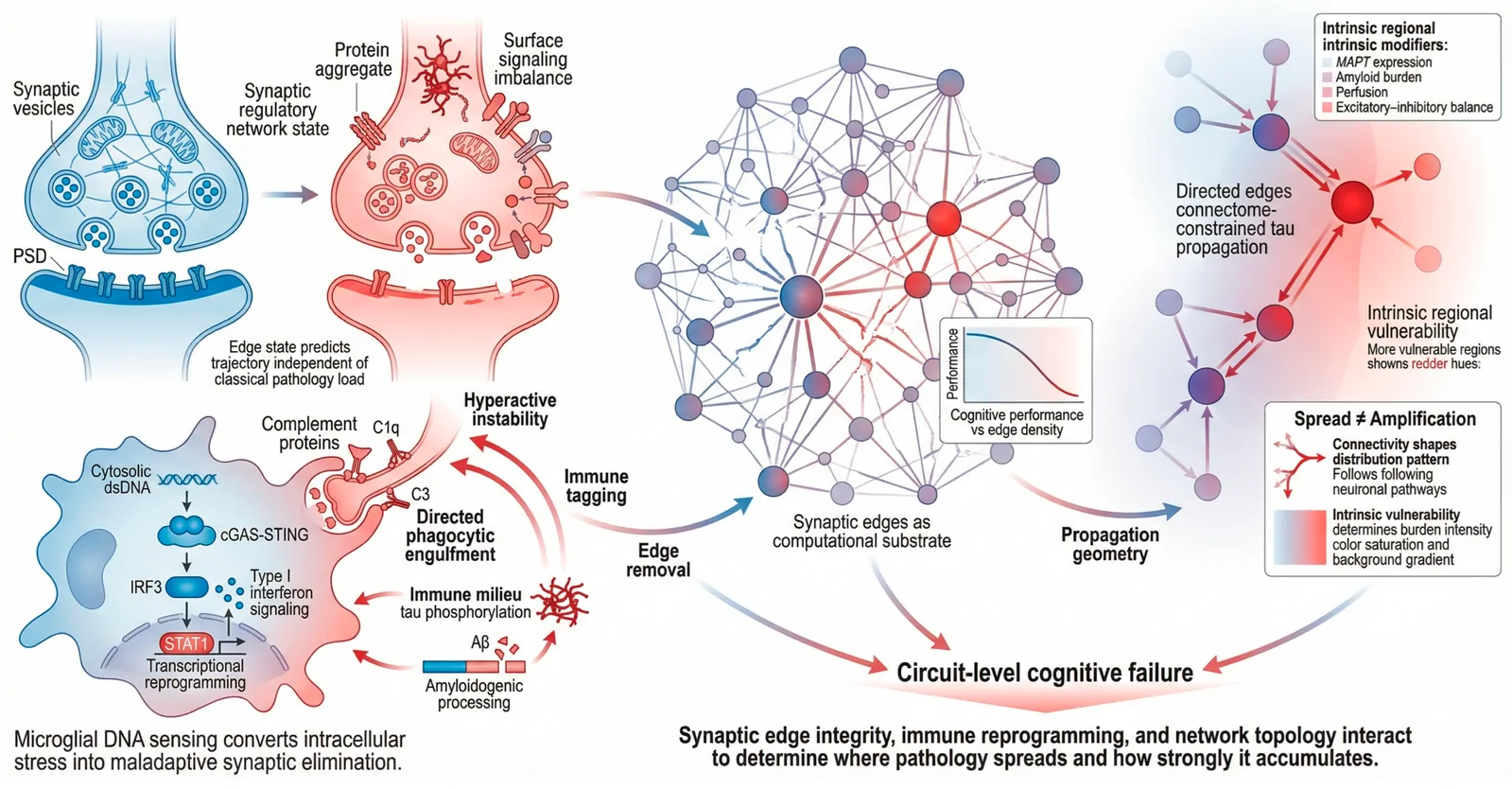
Immune-gated synaptic edge failure and topology-conditioned network destabilization in AD. Synaptic destabilization shifts network edges from homeostatic to maladaptive states. Microglial cGAS–STING activation induces interferon signaling and complement-mediated pruning (C1q/C3), promoting selective elimination of vulnerable synapses. Hub-associated edge loss disproportionately disrupts network integrity, producing nonlinear fragility. Connectome structure shapes propagation geometry, whereas intrinsic regional modifiers determine amplification magnitude. Together, immune reprogramming and topology-conditioned edge removal link molecular stress to circuit-level cognitive decline.

These converging mechanisms are schematized in Figure 6, which integrates synaptic edge destabilization, immune-mediated pruning, and topology-conditioned propagation into a unified network-failure model.

Taken together, synaptic failure in AD reflects coordinated edge-level destabilization driven by proteostatic stress, cytoskeletal transport disruption, and glial reprogramming rather than isolated synapse loss. Collectively, immune-gated pruning, astrocyte-mediated coupling, and topology-dependent vulnerability link molecular dysfunction to progressive breakdown of circuit-level integration, as illustrated in Figure 5 and Figure 6.

## 4. Multi-Omics Systems Modelling Frameworks for Network Biology in Alzheimer’s Disease

Reframing AD as a multi-layer network disorder requires specifying the mathematical structure assumed to underlie the disease. Mechanistic interpretation in contemporary AD research no longer rests on individual molecular cascades but on how heterogeneous omics layers are integrated to reconstruct coordinated disease states. Large-scale AD multi-omics analyses demonstrate that combining genomics, transcriptomics, epigenomics, and proteomics improves classification and biomarker discovery compared with single-modality approaches, underscoring that no single layer captures disease structure in isolation [132].

Human brain multimodal atlases provide empirical grounding for this integrative requirement. Integrated single-nucleus transcriptomic and epigenomic profiling across 84 donors spanning the AD continuum revealed progressive, cell-type-specific alterations along a pseudoprogression axis, with distinct vulnerability patterns across neuronal and glial populations [133]. These structured but heterogeneous changes indicate that disease progression manifests as coordinated reorganization of cellular programmes rather than uniform molecular dysregulation. Multi-omic measurements, therefore, serve as complementary projections of a shared, inferred disease state.

At a preclinical scale, paired targeted plasma fatty-acid and spontaneous-behavior profiles resolved coordinated low-dimensional organization in healthy rats; under AlCl_3_ exposure, a related cross-domain design detected dose-related reorganization of fatty-acid, behavioral, and cholinergic readouts. These studies illustrate a tractable design for testing multivariate state shifts, but they do not establish AD-specific mechanisms or causal lipid–behavior relationships [134,135].

Different integration architectures formalize this state in distinct ways. Latent-factor and deep learning approaches compress cross-modal variation into low-dimensional representations associated with diagnosis and progression [132]. In the ROSMAP cohort, Tao et al. implemented a staged graph convolutional network with uncertainty quantification (SGUQ) that sequentially incorporated transcriptomic, DNA methylation, and miRNA data based on prediction confidence [136]. mRNA data alone achieved an accuracy of 0.80 and was sufficient for confident classification in 46.23% of cases; an additional 16.04% required integration of DNA methylation, while full tri-modal integration yielded an overall accuracy of 0.858, outperforming multiple established multi-view learning frameworks. Importantly, the staged design formalized modality contributions as conditional rather than simultaneous, demonstrating that multi-omics integration in AD can be structured around uncertainty-driven escalation rather than fixed-feature concatenation.

A concrete mechanistic instantiation of dependency-based modeling is provided by single-microglia transcriptomic transition network analysis integrating approximately 0.7 million human single-nucleus RNA sequencing (snRNA-seq) nuclei across multiple brain regions [137]. Using a variational autoencoder for cross-cohort integration and transfer entropy–based gene regulatory inference constrained by the human protein–protein interactome, the study reconstructed subtype-specific transition networks linking homeostatic microglia to disease-associated microglia (DAM), tau microglia, and neuroinflammation-like microglia (NIM). Topological analysis identified putative molecular drivers—including SYK, INPP5D, CTSB, PRKCA, and ADAM10—within these transition modules, several of which overlap with established AD risk loci and microglial regulatory pathways. These transition networks were further used for hypothesis-generating drug prioritization, leading to the nomination of ketorolac as a repurposable candidate. However, the supporting evidence remains indirect, comprising computational network-based inference, transcriptomic observations in patient-derived microglial models, and retrospective real-world association analyses, rather than prospective mechanistic or interventional validation.

Within this context, the network-informed framework extends beyond conventional single-pathway and multi-target-directed strategies, which are typically organized around predefined targets within known biochemical pathways. In contrast, multi-omics and single-cell studies in Alzheimer’s disease indicate that pathology is structured through dynamic, cell-type–specific networks and state-dependent transitions [47,50,55,133]. Molecular drivers such as SYK, INPP5D, and CTSB therefore represent regulatory components within microglial transition networks rather than isolated pathway components [137], thereby enabling prioritization of intervention points that modulate disease-relevant state transitions rather than individual pathways [136,137,138].

Consistent with this perspective, recent AD-specific studies demonstrate that such computational frameworks integrate transcriptomic and proteomic signatures with drug perturbation profiles to construct drug–disease interaction networks and apply network proximity or signature-reversal metrics for candidate prioritization [139]. Complementary network-medicine approaches extend this paradigm by combining multi-dimensional network scoring with pharmacological and physicochemical constraints to prioritize clinically tractable candidates within disease-relevant interactome modules [140].

Temporal reconstruction further demonstrates that “disease stage” is a model-dependent construct rather than a directly observed biological state. In a systematic comparison of multi-state models fitted to six independent AD cohorts, Birkenbihl et al. showed that estimated transition probabilities, sojourn times, and hazard ratios differed significantly across datasets, even when evaluated on identical participant profiles. Models trained on individual cohorts learned cohort-specific covariate effects that propagated into divergent progression trajectories, and naive pooling biased estimates toward the largest sample. These findings indicate that inferred disease dynamics reflect the statistical distribution from which the model is trained, rather than a universally stable progression pathway [141].

Comprehensive AD-focused guidelines for single-cell sequencing analysis show that preprocessing thresholds, normalization models, dimensionality reduction choices, clustering strategies, and regulatory network inference pipelines materially alter cell-type definitions, trajectory reconstruction, and gene interaction maps. By implementing and benchmarking analytic workflows on AD snRNA-seq datasets, Wang et al. demonstrate that downstream biological interpretation is sensitive to computational design decisions [138].

**Figure 7:**
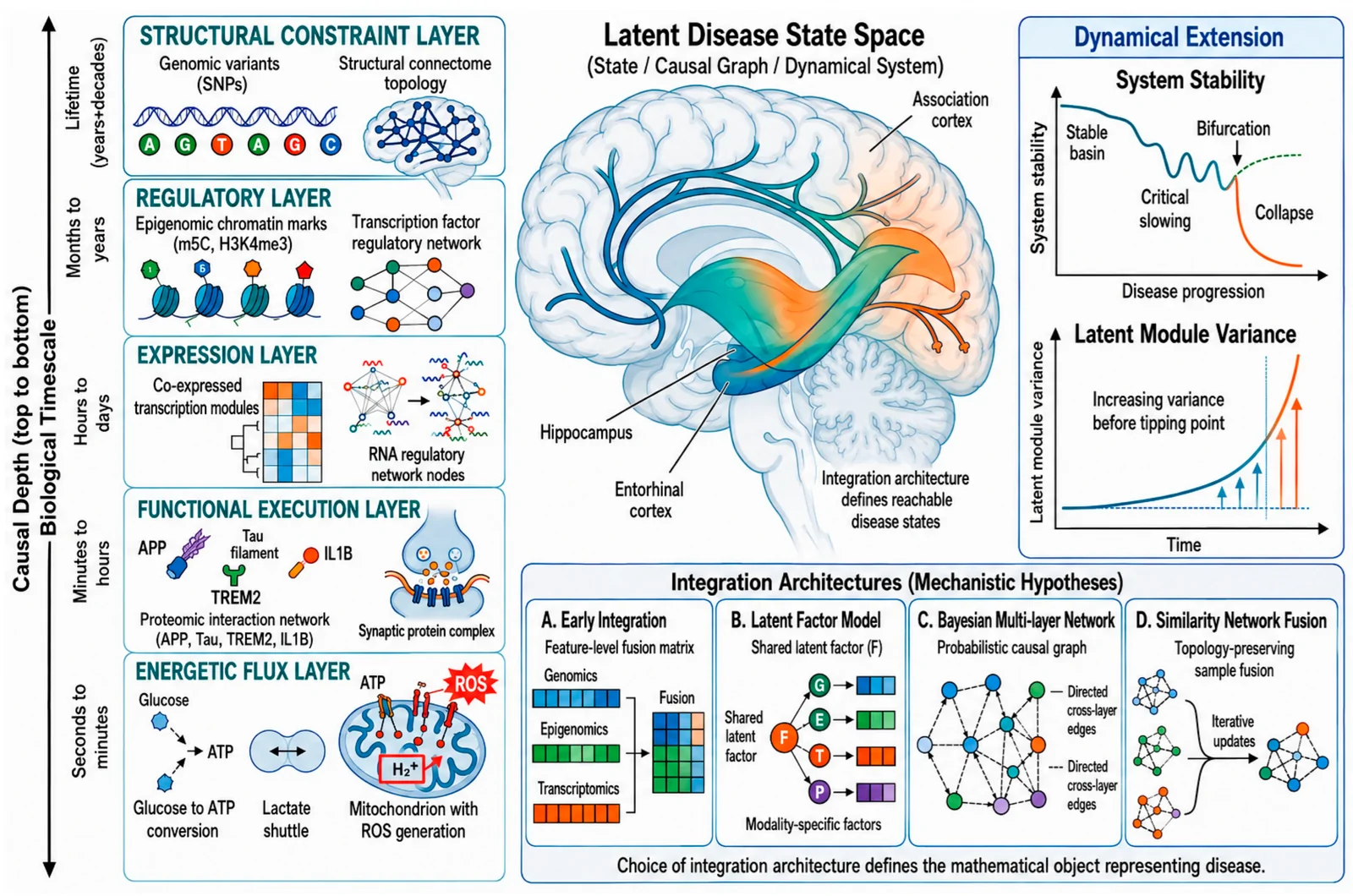
Multi-omic AD as a hierarchically embedded dynamical system across biological timescales. Layers are organized by causal depth and characteristic timescale, from lifetime-scale genomic constraints (SNPs, connectome topology) to months–years of regulatory and transcriptomic processes, to minutes–hours of proteomic and synaptic activity, and to seconds–minutes of metabolic flux. These layers project onto a latent disease state space in the brain. Alternative multi-omics integration architectures and dynamical extensions illustrate distinct modeling strategies and potential stability transitions.

As summarized in Figure 7, latent-factor models, staged multi-view architectures, dependency graphs, and progression models each instantiate a distinct mathematical object representing disease.

Among these, latent-factor models formalize integration by decomposing cross-modal variation into a reduced set of latent variables representing shared and modality-specific structure. In AD, such approaches capture coordinated molecular programmes that track neuropathology and cognitive decline across individuals, effectively summarizing high-dimensional data into biologically interpretable axes of variation [33]. However, because these latent variables are derived from covariance structure, they do not encode directionality or interaction flow between biological layers, and therefore remain descriptive representations of system state rather than mechanistic models of causal propagation [33].

In contrast, staged multi-view frameworks formalize multi-omics integration as a sequential, decision-driven process in which modalities are incorporated conditionally based on predictive uncertainty. In the SGUQ model applied to AD, transcriptomic data alone achieved 0.80 accuracy and resolved 46.23% of cases, while DNA methylation and miRNA layers were introduced only for samples with higher uncertainty, increasing overall accuracy to 0.858 [136]. This conditional escalation demonstrates that modality ordering reflects information gain and classification confidence rather than biological hierarchy, indicating that these frameworks are optimized for prediction and resource allocation rather than for representing mechanistic dependencies between molecular layers [142].

Dependency-based and transition-network models move closer to mechanistic interpretation by explicitly modeling structured relationships, temporal ordering, and directional information flow between molecular entities and cellular states [137]. In AD, single-cell analyses integrating trajectory inference with transfer entropy–based gene regulatory modeling reconstruct transitions between microglial states and identify central drivers such as SYK, INPP5D, and CTSB within these networks [137]. These approaches therefore capture state transitions and regulatory influence within evolving cellular systems. However, the inferred directionality reflects statistical dependencies constrained by observational data rather than experimentally validated causal mechanisms and should therefore be interpreted as causality-approximating rather than definitive [137]. This limitation is further reinforced by comparative analyses of AD progression models across independent cohorts, which demonstrate that estimated transition probabilities and disease trajectories vary substantially depending on dataset-specific distributions [141]. These findings indicate that model-derived dynamics are not universally stable biological pathways but context-dependent representations, placing inherent constraints on causal interpretation from observational multi-omics data alone.

Taken together, these distinctions define a hierarchy of inference in which latent-factor models describe coordinated system states, staged multi-view frameworks optimize predictive integration without mechanistic ordering, and dependency-based models provide the most suitable framework for mechanistic hypothesis generation in AD, while remaining complementary to latent-state representations that capture coordinated system-level organization. This hierarchy aligns with the progressive, state-dependent, and cell-type–resolved nature of the disease, in which biological interpretation requires preservation of structured dependencies and temporal dynamics across molecular and cellular layers.

Recent evidence from integrated omics and neurobiology now establishes that these architectures unfold across nested biological timescales. Stable genomic variants such as *APOE*, *GSAP*, and *FAM222A* define lifelong structural predispositions that shape connectomic and metabolic vulnerability [143,144]. Over months to years, gradual epigenomic remodeling—including gains in H3K27ac and H3K9ac and progressive DNA-methylation divergence—reshapes transcriptional accessibility and immune–neuronal coupling [145,146]. Transcriptomic dynamics fluctuate over hours to days, linking neuronal stress and glial activation modules across tissues [147,148]. Proteomic interactions, including modulation of the APP–TREM2 complex and phosphorylation signaling, occur on the order of minutes to hours [149]. Finally, mitochondrial and redox metabolism exhibit second-to-minute fluxes in ATP and ROS dynamics during bioenergetic stress [150].

Multi-omic integration in large AD cohorts further shows that these fast and slow processes are temporally coupled, forming hierarchical feedback loops in which metabolic and immune modules interact across regulatory, transcriptomic, proteomic, and cellular layers [50]. Overall, multi-omics modeling frameworks indicate that AD is best understood as a dynamically evolving system in which heterogeneous data layers represent complementary projections of a shared latent disease state. Collectively, differences in integration architecture and temporal scale define distinct representations of this state, reinforcing that disease progression emerges from coordinated, cross-scale network dynamics rather than fixed molecular pathways.

## 5. Artificial Intelligence for Network Reconstruction and Stratification in Alzheimer’s Disease

Within the framework of AD as a multi-layer network disorder, artificial intelligence (AI) is most rigorously positioned as a computational strategy for reconstructing and integrating disease-associated molecular networks across biological scales. Multi-omics investigations in AD consistently demonstrate that genetic risk, transcriptomic dysregulation, proteomic alterations, and neuroimaging phenotypes are not independent features but components of interacting molecular and systems-level networks [23,34]. These studies emphasize that machine learning is required not merely for classification, but for organizing heterogeneous data into structured representations that preserve regulatory and pathway relationships relevant to AD pathobiology.

Direct empirical support for AI-mediated network reconstruction in AD comes from interpretable deep learning frameworks that integrate GWAS and transcriptomic data. Xu et al. (2022) developed a model that translated genetic associations and multi-omics signals into biologically interpretable subnetworks, identifying disease modules linked to known AD pathways and nominating candidate drug repurposing targets [151]. Crucially, the learned latent structures corresponded to coherent molecular interaction modules rather than isolated gene rankings, demonstrating that AI can recover network-level architecture embedded within AD genomic data.

Beyond single-layer molecular inference, multiscale network modeling has shown that AI can link molecular dysregulation to higher-order phenotypes in AD. A recent study integrating transcriptomic, imaging, and cognitive data constructed cross-scale networks and identified brain hypometabolism as a central hub connecting molecular perturbations with clinical manifestations [152]. This work provides concrete evidence that network-based computational modeling can capture propagation of dysfunction across biological layers, consistent with the conceptualization of AD as a coupled multi-layer system. Graph-based multi-omics integration further refines this systems-level approach by explicitly embedding interaction topology into model structure. Dong and Zhong (2025) reviewed proteomics-driven integration strategies in AD, highlighting graph-constrained and deep learning methods that preserve protein–protein and pathway relationships during inference [153]. Similarly, Xu et al. (2025) introduced an explainable multi-omics integration framework based on graph representation learning that improves disease subtyping while retaining pathway-level interpretability in neurodegenerative datasets [154].

These topology-aware approaches are particularly suited to AD, where high dimensionality and limited sample sizes necessitate biologically constrained inference to avoid spurious associations. More broadly, AI-assisted analysis across individual omics layers, including transcriptomics and lipidomics, can facilitate biomarker discovery, molecular-pattern and pathway-level interpretation, and the translation of high-dimensional molecular profiles toward precision-medicine applications [155,156].

Collectively, AI-based approaches in AD function as network-reconstruction frameworks that integrate multi-omics data into biologically structured representations rather than isolated predictive features. These methods enable identification of disease-relevant modules, cross-scale interactions, and patient-specific network states. In therapeutic development, the same representations may support the nomination of druggable control nodes, the ranking of repurposing candidates, the identification of biomarker-enriched trial populations, and the evaluation of treatment-effect heterogeneity. More broadly, the translation of data-intensive, AI-assisted, and multi-omics approaches into precision medicine requires careful consideration of data quality and representativeness, interpretability, privacy, ethical governance, and clinical generalizability, particularly when heterogeneous molecular and phenotypic information is integrated for patient stratification [157,158,159]. Nevertheless, these outputs remain hypothesis-generating and require prospective perturbational and clinical validation before they can support treatment selection.

## 6. From Network Reconstruction to Causal and Translational Insight in Alzheimer’s Disease

**Figure 8:**
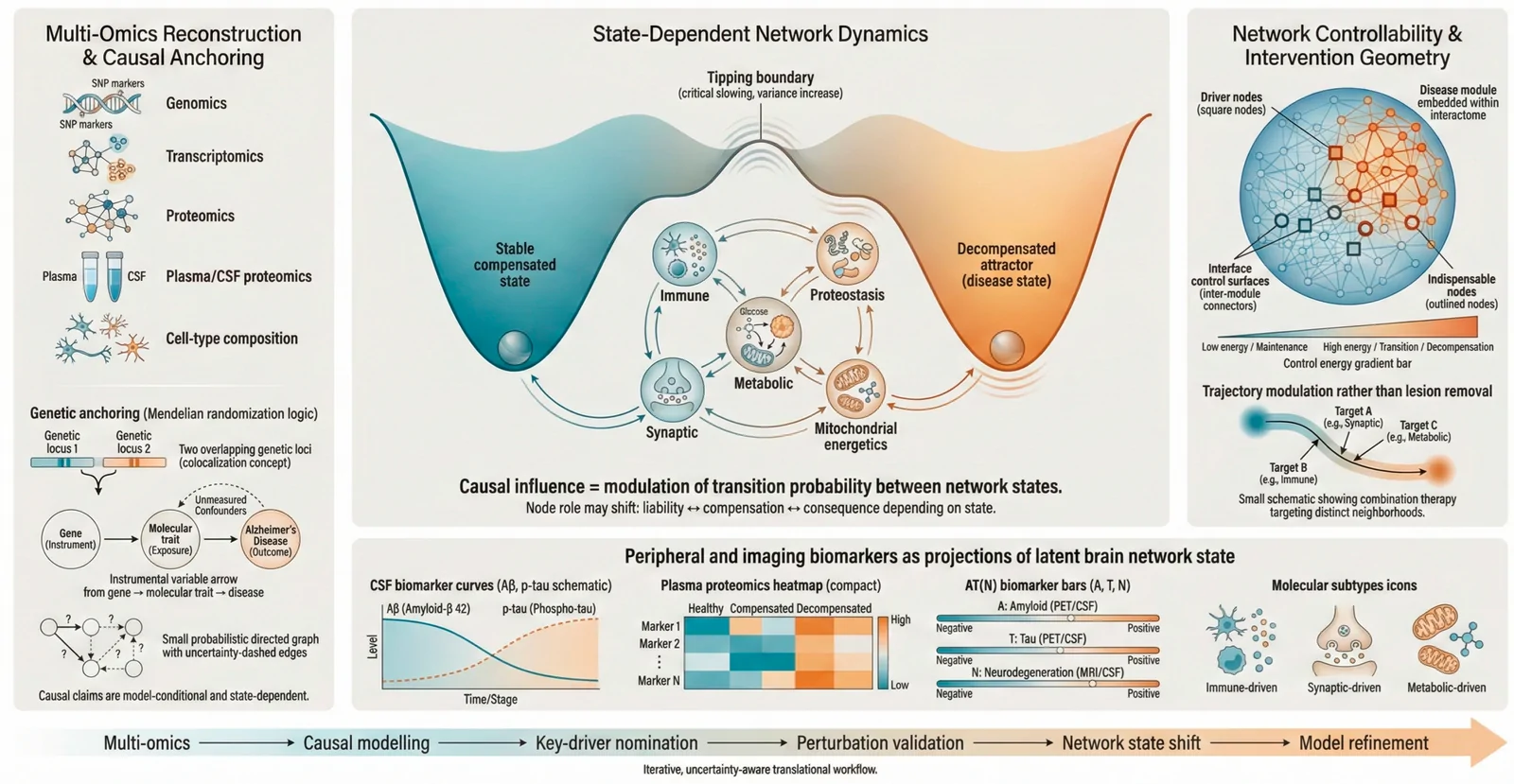
Conceptual framework linking network reconstruction to disease-state transitions in AD. The figure summarizes a systems-level view in which AD emerges from coordinated reorganization of molecular and cellular processes within a single integrated disease system rather than from a single linear cascade. Multi-omics data are integrated to infer latent network structure and state-dependent dynamics, capturing how adaptive configurations can maintain function despite accumulating pathology and how loss of resilience leads to disease expression. In this framework, causality reflects context-dependent influence on system behavior, biomarkers represent partial projections of underlying network states, and therapeutic action is framed as modulation of disease trajectories rather than correction of isolated lesions.

Recent AD systems biology studies have clarified how inherited genetic risk is embedded within cell-type–specific regulatory programmes and how these programmes reorganize across disease stages. The conceptual structure summarized in Figure 8 reflects this evidence-based progression from genetic localization to multi-layer molecular reconfiguration.

Genome-wide association studies have demonstrated that risk loci for late-onset AD are enriched in genes involved in immune response, lipid metabolism, and endosomal processes [43,44]. Both studies performed pathway enrichment analyses and reported overrepresentation of risk-associated variants within immune-related pathways. Bellenguez et al. further reported enrichment of risk gene expression in microglia relative to other brain cell types using single-cell reference datasets. These findings localize inherited susceptibility to defined biological processes and cellular contexts [43].

Cell-type–specific regulatory mapping provided additional mechanistic resolution. Nott et al. (2019) generated enhancer–promoter interaction maps from purified human brain cell populations and demonstrated that AD-associated variants are significantly enriched within microglial enhancers [160]. By linking noncoding risk variants to target genes through chromatin interaction data, this study provided direct evidence that genetic risk localizes to regulatory elements active in microglia.

Integration of genetic and proteomic data has further examined how inherited risk relates to protein abundance. Wingo et al. (2021) performed proteome-wide association analyses using human brain proteomic datasets and GWAS summary statistics [161]. They identified proteins whose genetically predicted abundance was associated with AD risk, thereby implicating protein-level variation in disease susceptibility. Large-scale quantitative proteomic profiling of human AD cortex demonstrated coordinated alterations in protein co-expression modules enriched for synaptic, mitochondrial, and immune-related proteins [74]. Rather than isolated protein abundance differences, the study identified structured module-level changes associated with neuropathological measures, including β-amyloid and tau burden. These findings indicate that proteomic reorganization in AD occurs within coordinated molecular networks.

Single-cell and single-nucleus studies have shifted the interpretation of AD from bulk tissue dysregulation toward cell-type–restricted regulatory reprogramming. In the prefrontal cortex, Mathys et al. (2019) showed that transcriptional changes differ not only by cell type but by pathological stage, with distinct expression programmes emerging in early versus late AD [162]. These findings demonstrate that molecular alterations are not uniformly distributed across cellular compartments and that transcriptional organization varies with disease severity. Grubman et al. (2019) independently reported cell-type–specific transcriptional signatures in entorhinal cortex—an early-affected region—indicating that vulnerability is regionally and cellularly structured [163]. Extending this framework, Morabito et al. (2021) integrated single-nucleus chromatin accessibility with transcriptomics and identified disease-associated shifts in regulatory element accessibility in microglia and oligodendrocytes [49]. The convergence of transcriptional and epigenomic alterations within defined cell populations indicates that AD progression involves coordinated remodeling of regulatory landscapes rather than isolated gene expression changes.

Causal interpretation of AD risk loci has been strengthened by statistical colocalization analyses integrating GWAS and eQTL datasets. By applying Bayesian colocalization across 109 expression datasets, Schwartzentruber et al. (2021) identified loci in which AD association signals and gene expression variation share a high posterior probability of a common causal variant [164]. These analyses enabled probabilistic prioritization of candidate genes within risk loci and demonstrated that strong colocalization evidence is enriched among independently supported AD genes, refining inference beyond proximity-based annotation.

Temporal staging provides an external anchor for these molecular dynamics. The NIA-AA framework established that amyloid deposition typically precedes tau-related neurodegeneration [2], while multi-omics integration has documented heterogeneity in molecular signatures among individuals with comparable biomarker profiles [34].

Together, these observations imply that biomarker-defined disease categories correspond to multiple underlying molecular configurations rather than a single uniform state.

Importantly, converging conceptual models of AD indicate that molecular configurations can be organized into distinct state-dependent regimes that differ in their capacity to buffer pathology. The mitochondrial cascade hypothesis explicitly distinguishes a phase of “compensated brain aging,” in which mitochondrial reserve capacity, metabolic flexibility, and synaptic adaptation maintain functional stability despite accumulating pathology, from an “uncompensated” phase in which exhaustion of bioenergetic and proteostatic buffering coincides with the emergence of clinical AD [165,166,167]. Within this framework, early amyloid and related biomarker changes are not sufficient to drive symptoms in isolation but may remain functionally tolerated until compensatory capacity declines below a critical threshold [165,167].

Complementary resilience-based and systems-level models extend this distinction beyond metabolism, demonstrating that individuals can sustain cognition despite substantial amyloid and tau pathology through preserved network redundancy, synaptic reserve, and adaptive plasticity, whereas symptomatic disease reflects a transition to a decompensated state marked by loss of resilience and accelerated decline [168,169,170]. Consistent with this view, amyloid–tau biomarker positivity does not uniquely define disease severity, and longitudinal multi-state models demonstrate that the probability of progression between clinically defined AD stages varies markedly with disease stage and patient characteristics, with substantially higher transition probabilities observed in earlier disease phases. Such heterogeneity in progression dynamics provides a quantitative basis for stage-dependent therapeutic strategies and clinical trial design [171].

Overall, integrated genetic, regulatory, proteomic, and single-cell evidence indicates that AD progression reflects coordinated reorganization of cell-type–specific molecular networks rather than isolated pathological events. Collectively, these findings support a state-dependent and context-sensitive framework, providing a conceptual basis for understanding disease expression as transitions between network configurations with differing resilience.

Compensatory capacity will therefore be operationalized as a pathology-by-redundancy interaction: higher module redundancy should attenuate the association between amyloid, tau, and neurodegeneration burden and longitudinal synaptic or cognitive decline [35,90,91]. It will be considered clinically informative only if it improves prediction of stage transition beyond pathology burden, age, sex, education, APOE genotype, and baseline cognition [36,172].

Building on this perspective, target prioritization may be guided not solely by pathway association, but by the position of molecular and cellular elements within the multiscale, state-dependent organization of disease. In this context, elements involved in state transitions or cross-module interactions, including microglial regulatory processes, endolysosomal–proteostatic pathways, and astrocyte-associated modulation of amyloid–tau relationships, may represent functionally influential points through which localized perturbations could propagate across biological levels.

Similarly, therapeutic response may be more appropriately interpreted by distinguishing pathway engagement from broader patterns consistent with stabilization of disease-related system dynamics. Changes in single-molecular readouts, such as amyloid reduction alone, may be insufficient to capture overall disease behavior. Instead, patterns such as attenuation of amyloid–tau coupling, modulation of glial-associated signatures, preservation of synaptic-related biomarkers, and slower progression across multidimensional biomarker profiles may provide complementary indications of coordinated changes across biological levels, while remaining subject to further empirical validation.

## 7. A Unified Model of Progressive Network Destabilization in Alzheimer’s Disease

A coherent disease trajectory emerges when AD is interpreted through longitudinal biomarker data rather than isolated pathway models. Contemporary research frameworks define AD biologically—by amyloid, tau, and neurodegeneration biomarkers—independent of clinical syndrome, reflecting the consistent observation that molecular pathology precedes symptoms by many years [173,174]. Longitudinal studies in autosomal dominant AD demonstrate that amyloid accumulation begins approximately 15–20 years before expected symptom onset, followed by tau deposition in medial temporal regions, subsequent neocortical tau spread, hippocampal atrophy, and only later measurable cognitive decline [175]. Importantly, similar biomarker trajectories are observed in sporadic late-onset AD, indicating a shared biological cascade across disease forms [176].

This temporal dissociation between pathology accumulation and clinical expression explains why substantial amyloid burden may coexist with preserved cognition: amyloid deposition alone does not tightly track symptom progression. In contrast, tau pathology and neurodegeneration show stronger alignment with cognitive decline. Entorhinal tau burden predicts subsequent neocortical spread and future cognitive deterioration [175], and combined amyloid plus phosphorylated tau biomarkers outperform amyloid alone in predicting progression [177]. Rates of hippocampal atrophy and cognitive decline accelerate after symptomatic onset in both familial and sporadic AD [176], indicating nonlinear acceleration rather than constant deterioration.

From a systems perspective, biomarker relationships are not strictly linear. Large-scale cerebrospinal fluid profiling demonstrates that the relationship between amyloid and tau follows nonlinear, profile-dependent dynamics, with individuals transitioning between biomarker-defined states over time [178]. Plasma-based AT(N) classification further demonstrates that AD dementia (clinical stages 4–6) risk is not uniformly distributed across biomarkers but is phenotype-specific. In a prospective midlife cohort, isolated tau positivity (T+) was associated with markedly increased dementia risk (adjusted risk ratio [aRR] = 4.60), whereas the combined amyloid plus neurodegeneration profile (A+N+) independently conferred elevated risk (aRR = 2.70), even when amyloid alone did not [179]. Overlapping categories such as A+T+N+ and T+N+ occurred more frequently than expected by chance, indicating non-random biomarker convergence. These observations support the interpretation that progression involves structured transitions across multidimensional biomarker states rather than monotonic accumulation of a single lesion.

**Figure 9:**
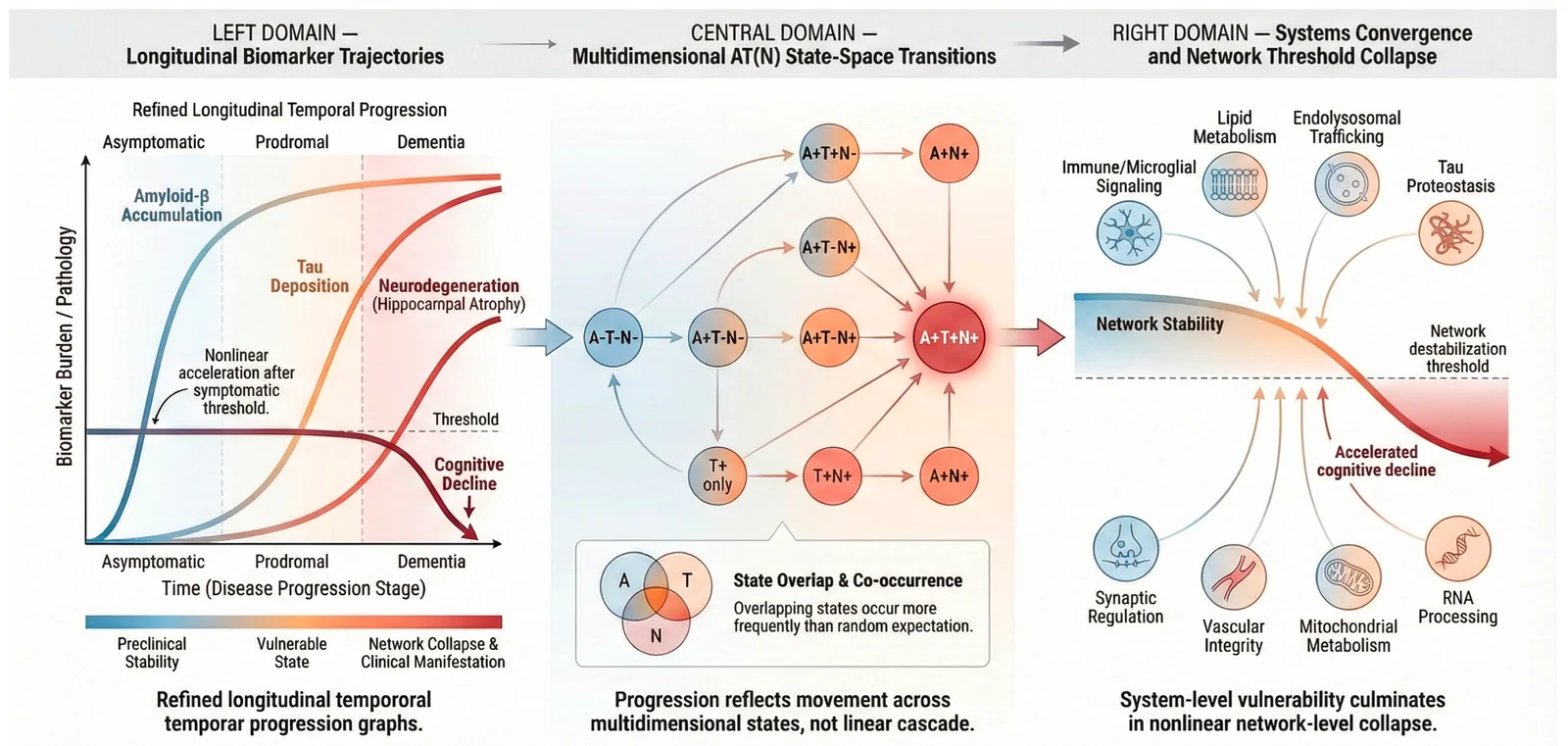
Conceptual model of progressive network destabilization in AD. This figure presents a conceptual systems-level synthesis of longitudinal biomarker evidence in AD. The left panel illustrates the temporal sequence of biomarker changes across the clinical continuum, with early β-amyloid (A) deposition, followed by aggregated tau pathology (T), subsequent neurodegeneration (N), and nonlinear acceleration of cognitive decline after symptomatic threshold crossing. The central panel depicts progression within the NIA–AA AT(N) biomarker framework, in which A denotes biomarkers of β-amyloid deposition, T denotes biomarkers of aggregated tau pathology (neurofibrillary tangles), and (N) denotes biomarkers of neurodegeneration or neuronal injury that are not specific to AD pathologic change. Movement across multidimensional AT(N) states reflects structured biomarker convergence rather than a strictly linear cascade. The right panel integrates distributed biological vulnerability—including immune and microglial signaling, lipid metabolism, endolysosomal trafficking, tau proteostasis, synaptic integrity, vascular function, and mitochondrial metabolism—into a progressive loss-of-resilience framework. AD dementia (clinical stages 4–6) emerges when cumulative cross-system coupling exceeds compensatory capacity, resulting in a threshold-like transition from compensated network function to destabilized and accelerated decline.

This conceptual transition—from longitudinal biomarker trajectories to multidimensional state-space movement and eventual systems convergence—is schematically illustrated in Figure 9.

Genetic evidence indicates that AD does not arise from a single pathogenic cascade. More than 70 late-onset AD risk loci have been identified. Together, they implicate disruption across multiple interacting biological systems, including immune and microglial signaling, lipid homeostasis, endolysosomal trafficking and APP processing, tau proteostasis, synaptic regulation, vascular integrity, mitochondrial metabolism, and RNA splicing [43,180].

Beyond amyloid and tau, recent evidence highlights multiple non-amyloid/non-tau (NANT) biological processes—including neuroinflammation, synaptic dysfunction, vascular injury, and metabolic disruption—as integral components of AD pathophysiology. Several NANT biomarkers track neurodegeneration independently of amyloid status, while others vary across disease stage. Early-phase clinical trials targeting these pathways demonstrate measurable biomarker modulation and potential cognitive effects, supporting their incorporation into evolving diagnostic and therapeutic frameworks [181].

Collectively, current longitudinal and biomarker evidence supports a stage-dependent, nonlinear progression model in which early amyloid accumulation establishes biological vulnerability, tau propagation beyond medial temporal regions marks a transition toward accelerated neurodegeneration, biomarker coupling evolves in a state-dependent manner, and AD dementia (clinical stages 4–6) emerges when convergent tau-associated neurodegeneration and structural atrophy exceed compensatory capacity. While formal dynamical bifurcation analyses have not yet been empirically validated in human cohorts, observed acceleration points, biomarker state transitions, and network-structured spread are consistent with a threshold-like systems transition rather than a purely linear toxic accumulation model.

In summary, longitudinal and biomarker observations can be interpreted within a nonlinear and stage-dependent conceptual framework, in which disease-related changes are considered as potential transitions across multidimensional network states rather than sequential accumulation of isolated pathologies. Collectively, this perspective provides a conceptual basis for exploring how clinical expression may relate to progressive loss of system-level resilience in the context of converging molecular, cellular, and network-level alterations.

## 8. Therapeutic Implications: Current Interventions and Future Network-Guided Strategies

Within the proposed framework, interventions can be mapped according to the biological module engaged and the disease stage at initiation. Cholinesterase inhibitors and memantine provide symptomatic benefit but are not established disease-modifying therapies [7]. Lecanemab and donanemab lower cerebral amyloid burden and modestly slow clinical decline in selected patients with biomarker-confirmed early symptomatic AD [14,15], although their use requires careful eligibility assessment and MRI monitoring for amyloid-related imaging abnormalities (ARIA) [16,17]. Within this network interpretation, anti-amyloid antibodies primarily modify the amyloid–proteostatic layer; amyloid removal should not itself be interpreted as restoration of downstream tau, glial, vascular, or synaptic dysfunction.

Emerging therapies illustrate why target engagement must be distinguished from clinical benefit. In a phase 1b trial, MAPTRx (BIIB080), which promotes RNase H1-mediated degradation of MAPT pre-mRNA, produced dose-dependent reductions in cerebrospinal-fluid total tau and p-tau181, with greater than 50% mean total-tau reduction at the higher doses; the study was not designed to establish clinical efficacy [182]. In phase 2, gosuranemab lowered unbound cerebrospinal-fluid N-terminal tau without improving Clinical Dementia Rating–Sum of Boxes scores at 78 weeks [183]. Similarly, AL002 reduced cerebrospinal-fluid soluble TREM2 and increased osteopontin, indicating target engagement and pharmacodynamic activity, but did not meet its primary Clinical Dementia Rating–Sum of Boxes endpoint; ARIA-like MRI changes were the most frequent treatment-emergent adverse events [184]. These findings support combining module-specific target-engagement biomarkers with clinical and complementary downstream measures when evaluating whether a localized perturbation produces broader system-level effects.

Future development should test biomarker-gated sequential or combination approaches rather than assume that a uniform multitarget regimen will be appropriate across the AD continuum. The DIAN-TU Tau NexGen phase 2/3 platform evaluates etalanetug (E2814), an antibody directed against the microtubule-binding region of tau, alone and with background lecanemab across asymptomatic and symptomatic dominantly inherited AD cohorts (NCT05269394) [185]. This provides a clinical test of stage-dependent amyloid–tau intervention, although efficacy remains undetermined. Within the proposed framework, future trials could similarly evaluate glial, vascular, metabolic, or synaptic interventions in participants selected using biomarkers relevant to the targeted module.

Artificial intelligence and machine learning should be positioned as enabling methods rather than therapeutic interventions. AD-specific topology-aware and microglial transition-network models have been used to prioritize molecular targets and drug-repurposing candidates [137,151]; extending these approaches to longitudinal multimodal data could support future biomarker-defined trial stratification, but patient-level treatment selection has not been validated. Separately, an interpretable digital-twin trial method using retrospective trial-matched data reduced required sample sizes by 9.5–19.0% in CLARITY AD–parameterized simulations designed to detect a simulated 25% slowing of decline at 90% power, with type I error consistent with 0.05 [186]. This represents proof of concept for trial analysis and requires prospective validation before clinical implementation.

## 9. Conclusions

Despite major advances in biomarker discovery and multi-omics profiling, Alzheimer’s disease (AD) remains difficult to explain through pathway-centric or modality-isolated models, particularly regarding prolonged compensation, heterogeneous progression, and stage-dependent therapeutic response. This review extends existing network-medicine, connectomic, and multi-omics approaches by integrating three testable concepts: hierarchical constraint propagation, cross-scale buffering, and state-aware stratification. The proposed cross-layer relationships represent mechanistic hypotheses supported by convergent evidence rather than causal sequences established by every cited study.

The central synthesis is that biological layers—genomic, regulatory, transcriptomic, proteomic, metabolic, spatial, and connectomic—provide complementary measurements of an evolving disease state, whereas immune, lipid, endolysosomal, vascular, metabolic, and synaptic modules represent functional subsystems operating across these layers. Constraint propagation predicts that an upstream state should improve prediction of a subsequent downstream state beyond the downstream layer’s own history. Buffering capacity can be tested as a pathology-by-redundancy interaction, whereby greater independent-path redundancy attenuates the relationship of amyloid, tau, and neurodegeneration burden with longitudinal synaptic or cognitive decline. Candidate states such as compensated multi-module stress, inflammatory–metabolic amplification, and decompensated network failure provide corresponding hypotheses for research stratification.

Operationally, preclinical studies can perturb microglial–astrocytic, endolysosomal, metabolic, or synaptic modules and quantify whether effects propagate across subsequent molecular, cellular, and network layers. Longitudinal human studies can estimate independent-path redundancy, adjusted lagged cross-layer coupling, and piecewise function–pathology slopes, while testing whether plasma GFAP, vascular or blood–brain barrier measures, FDG-PET, CSF synaptic proteins such as YWHAG, and resting-state fMRI network segregation improve prediction of clinical-stage transition beyond conventional amyloid, tau, and neurodegeneration biomarkers and clinical covariates. Clinical trials may evaluate these composite states for research enrichment and use preservation of synaptic integrity, vascular function, metabolic activity, and network organization as complementary response endpoints. These applications are falsifiable research strategies requiring prospective multicohort and perturbational validation, not yet-validated diagnostic or treatment-selection rules.

## Figures and Tables

**Table 1:** Operational terminology used in this review.

Term	Operational Meaning
Layer	A biological organizational or measurement scale: genomic, regulatory/epigenomic, transcriptomic, proteomic/metabolic, cellular/spatial, connectomic, or clinical.
Module	A functionally coherent subsystem that may span layers, such as immune, lipid, endolysosomal, mitochondrial, proteostatic, cytoskeletal, synaptic, or vascular processes.
State	The joint configuration of measured layers and functional modules in an individual or model at a defined time.
Constraint propagation	A change in one layer that alters the range or probability of states accessible to another layer.
Buffering capacity/resilience	The ability of redundant or adaptive processes to preserve function despite perturbation or pathology.
Critical transition/tipping point	A candidate abrupt change in system state or coupling inferred from longitudinal dynamics; it remains a hypothesis unless demonstrated empirically.

**Table 2:** Empirical foundations and cross-layer links in the proposed AD framework. Representative human studies are shown unless experimental-model evidence is explicitly identified. The “Multi-Layer Framework Link” column identifies the represented layer and the downstream constraint proposed by the framework; it does not imply that the cited observational study alone establishes causality.

Evidence Domain	Representative Empirical Finding	Quantitative Anchor	Biological Implication for AD	Multi-Layer Framework Link	References
Genetic architecture	Late-onset AD loci converge on microglial/immune, lipid, endolysosomal, APP, and tau biology.	75 loci, including 42 newly associated loci; 1,126,563 individuals in a complementary GWAS	Inherited risk is concentrated in a limited set of stress-handling systems.	Genomic → regulatory: variants weight cell-type-specific enhancer and stress-response circuits.	[43,44,45,46]
Regulatory architecture	Risk variants are enriched in microglial enhancers, and chromatin accessibility is remodeled by cell type, region, and disease stage.	3.5 million cells; 384 brain samples; 6 regions; >1 million candidate cis-regulatory elements	Genetic risk is realized through cell-type- and stage-specific regulatory programmes.	Regulatory/epigenomic → transcriptomic: accessible enhancers delimit inducible gene-expression programmes.	[47,48,49]
Transcriptomic organization	Same-individual multi-omics resolves reproducible subtypes with distinct immune, metabolic, heat-shock, cytoskeletal, and synaptic programmes.	Up to 1358 brain samples; 11 subtypes; 3 AD-associated clusters	AD expression profiles span multiple molecular states rather than a single uniform signature.	Transcriptomic → proteomic/cellular state: expressed programmes determine protein production, turnover, and cell-state demands.	[50]
Proteomic network structure	Human brain and cerebrospinal fluid proteomics identify coordinated synaptic, mitochondrial, immune, and energy metabolism modules, with substantial RNA–protein discordance.	>2000 brains; nearly 400 cerebrospinal-fluid samples	Protein abundance and phosphorylation reveal realized network dysfunction not predictable from RNA alone.	Proteomic/phosphoproteomic → proteostatic/metabolic: protein states impose folding, degradation, signaling, and energetic load.	[51]
Endolysosomal and mitochondrial modules	Endolysosomal polygenic burden is associated with organelle enlargement, while mitochondrial dysregulation converges with inflammatory signaling.	13 endolysosomal-network loci; 1339 mitochondrial genes; 825 differentially expressed genes; 13 interaction pairs	Trafficking, degradation, and bioenergetic capacity are impaired in concert.	Organelle/cellular → proteostatic/metabolic: impaired clearance and ATP production restrict downstream cellular adaptation.	[8,52,53]
Lipid-associated architecture	Early-onset AD shares genetic covariance with lipid traits at APOE, TREM2, MS4A4E, LILRA5, and LRRC25, with additional prioritized genes.	AD: *n* = 19,668; lipid traits: *n* = 1,320,016; 5 shared loci; 3 covariance regions	Lipid handling is a shared inherited and metabolic determinant of AD vulnerability.	Genomic/metabolic → membrane/trafficking: lipid composition conditions membrane domains, endosomes, and proteolytic localization.	[54]
Spatial and clinical stratification	Multimodal profiles and multiregion regulatory states are associated with distinct decline, neurodegeneration, astrogliosis, and survival.	4 multimodal profiles; 384 brain samples; 6 regions	Molecular alterations are regionally structured and clinically heterogeneous.	Cellular/spatial → connectomic/clinical: local neighborhoods determine where dysfunction is amplified and how it reaches circuits.	[47,55]
Functional network architecture	Connectivity predicts pathology, dynamic brain states change by stage, and control-network connectivity modifies amyloid-associated decline.	*n* = 300; *n* = 239; *n* = 1021; 10 canonical networks; amyloid-by-connectivity interaction *p* = 0.025	Hub and edge integrity modulates how molecular pathology is expressed cognitively.	Connectomic → clinical: residual network integration constrains cognitive expression and transition to AD dementia (clinical stages 4–6).	[56,57,58,59,60]

## Data Availability

No new data were created or analyzed in this study. Data sharing is not applicable.

## Abbreviations

Aβ	Amyloid beta
AD	Alzheimer’s disease
AIF	Apoptosis-inducing factor
ALDH	Aldehyde dehydrogenase
APOE	Apolipoprotein E
APP	Amyloid precursor protein
ARIA	Amyloid-related imaging abnormalities
ARIA-E	Amyloid-related imaging abnormalities with edema/effusion
ARIA-H	Amyloid-related imaging abnormalities with hemosiderin deposition
AT(N)	Amyloid, Tau, Neurodegeneration biomarker framework
ATP	Adenosine triphosphate
BBB	Blood–brain barrier
BDNF	Brain-derived neurotrophic factor
Ca^2+^	Calcium ion
CNS	Central nervous system
CSF	Cerebrospinal fluid
DAMPs	Damage-associated molecular patterns
DEG	Differentially expressed gene
DNA	Deoxyribonucleic acid
ECM	Extracellular matrix
ER	Endoplasmic reticulum
ETC	Electron transport chain
fMRI	Functional magnetic resonance imaging
FTD	Frontotemporal dementia
GFAP	Glial fibrillary acidic protein
GWAS	Genome-wide association study
HIF-1α	Hypoxia-inducible factor 1 alpha
IFN	Interferon
IL	Interleukin
IGF2BP	Insulin-like growth factor 2 mRNA-binding protein
IPSCs	Induced pluripotent stem cells
ISR	Integrated stress response
LC3	Microtubule-associated protein 1A/1B-light chain 3
LOAD	Late-onset Alzheimer’s disease
LTP	Long-term potentiation
MAPK	Mitogen-activated protein kinase
MCI	Mild cognitive impairment
m6A	N6-methyladenosine
miRNA	MicroRNA
MRI	Magnetic resonance imaging
mtDNA	Mitochondrial DNA
mTOR	Mechanistic target of rapamycin
NAD^+^	Nicotinamide adenine dinucleotide
NFT	Neurofibrillary tangle
NF-κB	Nuclear factor kappa B
NLRP3	NOD-like receptor family pyrin domain containing 3
NVU	Neurovascular unit
PET	Positron emission tomography
PKA	Protein kinase A
PKC	Protein kinase C
p-tau	Phosphorylated tau
RNA	Ribonucleic acid
ROS	Reactive oxygen species
scRNA-seq	Single-cell RNA sequencing
snRNA-seq	Single-nucleus RNA sequencing
SOD	Superoxide dismutase
TCA	Tricarboxylic acid cycle
TDP-43	TAR DNA-binding protein 43
TREM2	Triggering receptor expressed on myeloid cells 2
UPS	Ubiquitin–proteasome system
VEGF	Vascular endothelial growth factor
WGCNA	Weighted gene co-expression network analysis
YTHDF2	YTH N6-methyladenosine RNA-binding protein 2
